# A Novel N-Terminal Pro-B-Type Natriuretic Peptide Assay in the Early Diagnosis of Acute Heart Failure

**DOI:** 10.1016/j.jacadv.2025.102206

**Published:** 2025-09-29

**Authors:** Maria Belkin, Desiree Wussler, Pedro Lopez-Ayala, Albina Nowak, Danielle M. Gualandro, Dilbar Sailova, Codruta Popescu, Ivo Strebel, Marie Niggemann, Julia Reinhardt, Gabrielle Huré, Nikola Kozhuharov, Freya Jenkins, Emel Kaplan, Fabiana Sgueglia, Zaid Sabti, Laureve Chollet, Katharina Rentsch, Joanna Gawinecka, Felix Mahfoud, Michael Christ, Angelika Hammerer-Lercher, Tobias Breidthardt, Christian Mueller

**Affiliations:** a Cardiovascular Research Institute Basel (CRIB), University Hospital Basel, University of Basel, Basel, Switzerland; b Department of Hematology, University Hospital Basel, University of Basel, Basel, Switzerland; c Department of Cardiology, Vancouver General Hospital, Vancouver, Canada; d Department of Cardiology, University Hospital Basel, University of Basel, Basel, Switzerland; e Department of Endocrinology and Clinical Nutrition, University Hospital Zurich, Zurich, Switzerland; f Division of Internal Medicine, University Psychiatry Clinic Zurich, Zurich, Switzerland; g Department of Cardiology, Inselspital Bern, Bern, Switzerland; h Department of Laboratory Medicine, University Hospital Basel, University of Basel, Basel, Switzerland; i Department of Laboratory Medicine, University Hospital Zürich, University of Zürich, Zürich, Switzerland; j Department of Emergency Medicine, Kantonsspital Luzern, Luzern, Switzerland; k Department of Laboratory Medicine, Kantonsspital Aarau, Aarau, Switzerland; l Department of Laboratory Medicine, Central Medical Laboratory, Feldkirch, Austria; m Department of Internal Medicine, University Hospital Basel, University of Basel, Basel, Switzerland

**Keywords:** acute heart failure, emergency department, NT-proBNP, NT-proBNP-Access assay

## Abstract

**Background:**

The performance of the novel NT-pro-B-type natriuretic peptide (NT-proBNP)-Access assay in the early diagnosis of acute heart failure (AHF) is unknown.

**Objectives:**

The objective of the study was to assess the diagnostic accuracy of NT-proBNP-Access in patients presenting with acute dyspnea and compare it to the established NT-proBNP-Elecsys assay.

**Methods:**

In a prospective multicenter diagnostic study enrolling patients presenting with acute dyspnea to the emergency department, NT-proBNP-Access was measured in a blinded fashion and compared to NT-proBNP-Elecsys concentrations. The primary endpoint was diagnostic accuracy quantified by area under the receiver operating characteristics curve (AUC). Secondary endpoints were the performance of the guideline-recommended clinical decision values (rule-out: <300 pg/mL, rule-in: age-adjusted >450/900/1,800 pg/mL) for AHF.

**Results:**

Among 1,400 patients (53% AHF), the NT-proBNP-Access assay yielded significantly higher NT-proBNP concentrations vs the NT-proBNP-Elecsys assay (median 2,087 pg/mL vs 1,568 pg/mL [*P* < 0.001]). The NT-proBNP-Access assay had very high diagnostic accuracy (AUC: 0.914; 95% CI: 0.898-0.93), which was slightly lower than the NT-proBNP-Elecsys assay (AUC: 0.922; 95% CI: 0.908-0.937; *P* = 0.006). Using guideline-recommended clinical decision values, the NT-proBNP-Access assay ruled out fewer patients compared to NT-proBNP-Elecsys (18.7% vs 26.3%) with similar sensitivity (98.9% vs 98.5%). Conversely, more patients were ruled in (58.1% vs 52.1%), with lower specificity (77.6% vs 84.8%; *P* < 0.001). Diagnostic concordance was high (85.3%), with major mismatch (no AHF vs AHF) uncommon (0.6%), but minor mismatch (gray zone vs rule in/rule out and vice versa) common (14.1%).

**Conclusions:**

The NT-proBNP-Access assay had a very high diagnostic accuracy for AHF. Levels were approximately 25% higher with NT-proBNP-Access vs NT-proBNP-Elecsys, resulting in minor diagnostic discordance in 1 of 7 patients using guideline-recommended decision values.

Acute heart failure (AHF) is one of the most common diagnosis in the emergency department (ED) requiring hospitalization^1^, ^2^, ^3^ and remains associated with unacceptably high morbidity and mortality.^1^^,^^2^^,^^4^ The dismal outcomes of patients with AHF may be partially attributed to diagnostic uncertainty in the ED, which delays both diagnosis and initiation of effective treatment.^3^^,^^5^, ^6^, ^7^, ^8^

Natriuretic peptides (NPs) are quantitative plasma biomarkers indicative for the presence and severity of hemodynamic cardiac stress and heart failure. Their use has substantially improved the rapid detection of AHF in patients presenting with acute dyspnea.^9^, ^10^, ^11^, ^12^, ^13^ Accordingly, the diagnostic use of NP has received a Class I recommendation in clinical practice guidelines.^1^^,^^2^ Current European Society of Cardiology and American Herat Association/American College of Cardiology/Heart Failure Society of America guidelines recommend the use of specific clinical decision values achieving excellent performance characteristics for the early rule out and/or rule in of AHF.^1^^,^^2^^,^ These clinical decision values were derived using the dominant N-terminal pro-B-type NP (NT-proBNP) assay in the current clinical use (NT-proBNP-Elecsys).^1^^,^^2^^,^^9^, ^10^, ^11^, ^12^, ^13^ Recently, novel NT-proBNP assays using different epitopes and/or antibody combinations including the NT-proBNP-Access assay have recently become clinically available. However, the diagnostic accuracy and the performance of guideline-recommended decision values using the novel NT-proBNP-Access assay in the early diagnosis of AHF remain unknown.

This study aimed to address this gap in knowledge by evaluating the following: 1) the diagnostic accuracy of the NT-proBNP-Access assay in direct comparison with the NT-proBNP-Elecsys; and 2) the performance of guideline-recommended decision values using the novel NT-proBNP-Access assay for the early diagnosis of AHF in a large multicentre diagnostic study using central adjudication of AHF.

## Methods

### Study population and design

The BASEL V (Basics in Acute Shortness of Breath EvaLuation; NCT01831115) was a prospective, diagnostic, and prognostic study which aimed at advancing the early detection and management of patients with acute dyspnea.^14^, ^15^, ^16^, ^17^ Adult patients presenting to the ED of 2 Swiss University Hospitals (Basel and Zurich) with acute dyspnea as their chief complaint were enrolled. Although enrollment was independent of renal function, patients with end-stage renal failure on chronic kidney replacement therapy were excluded. For this analysis, only patients whose NT-proBNP concentrations were measured by both the Elecsys assay and the Access assay from blood samples drawn on presentation to the ED were included. Patients were also excluded if the final diagnosis of acute dyspnea remained unclear.

The study adhered to the principles of the Declaration of Helsinki and was approved by the local ethics committees. All patients provided written informed consent. Reporting is according to the Strengthening the Reporting of Observational Studies in Epidemiology guidelines for cohort studies (Supplemental Table 1).

### Adjudication of the final diagnosis

Two independent cardiologists/internists adjudicated the final diagnosis using all available clinical information, including clinical history, physical examination, 12-lead electrocardiogram, laboratory findings, chest X-ray, echocardiography, lung function testing, computed tomography, the response to therapy, and autopsy data for patients who died in hospital. These findings included one of the NPs (BNP or NT-proBNP) that current guidelines recommended for diagnosing AHF with a class I recommendation.^1^^,^^2^ At the time of enrollment, Basel used a BNP assay (Axsym or Architect BNP, Abbott Laboratories), whereas Zurich used the NT-proBNP-Elecsys assay (Elecsys proBNP II, Roche Diagnostics AG) for routine NP measurements. In situations of diagnostic disagreement, cases were reviewed and adjudicated in conjunction with a third cardiologist/internist.

### Biochemical measurements of NT-proBNP

At ED presentation, blood samples were collected in tubes containing potassium ethylenediaminetetraacetic acid. Following centrifugation, samples were frozen at −80 °C for later batch analysis. Plasma NT-proBNP concentrations were measured in a blinded fashion using both the NT-proBNP-Elecsys assay and the NT-proBNP-Access assay (Beckman Coulter). Both assays use immunoassay techniques to quantify NT-proBNP concentrations. Although both target similar epitope ranges, they use distinct antibodies, detecting NT-proBNP at different epitopes. The Elecsys assay uses the electrochemiluminescence immunoassay technology, following a sandwich immunoassay method. In this method, 2 monoclonal antibodies target epitopes on the N-terminal and C-terminal regions of NT-proBNP. The detection process involves the formation of a sandwich complex and a chemiluminescence reaction, which is quantified by a photomultiplier. The test can be conducted on various cobas analyzers. This assay has a measurement range of 5 to 35,000 pg/mL, with possible extension to 70,000 pg/mL with dilution. For analytical sensitivity, the limit of blank is 3 pg/mL, limit of detection is 5 pg/mL, and limit of quantification is 50 pg/mL. The intra-assay precision ranges from 1.8% to 2.7% and total precision from 2.4% to 3.2%.^18^ The NT-proBNP-Access assay also uses a 1-step, 2-site sandwich immunoassay with chemiluminescent detection. A monoclonal anti-NT-proBNP antibody and a monoclonal anti-NT-proBNP antibody conjugated to alkaline phosphatase are added to the serum or plasma sample. The NT-proBNP in the sample binds to the solid-phase antibody, while the conjugated antibody binds to a separate antigenic site on the NT-proBNP molecule. Light generated by a chemiluminescent substrate is measured with a luminometer. The test is conducted by the automated Dxl 9000 Access Immunoassay Analyzer. The measurement range of the NT-proBNP-Access assay extends from 10 to 35,000 pg/mL, and after automated dilution, up to approximately 350,000 pg/mL. The limit of blank is 1.1 pg/mL, and both the limit of detection and limit of quantification are ≤10 pg/mL, ensuring consistent detection and quantification of low NT-proBNP concentrations. Across multiple sites and days, results showed strong consistency: within-run variability ranged from 1.4% to 4.9%, whereas day-to-day and site-to-site variability remained below 2.0%. Overall reproducibility ranged from 3.0% at lower concentrations to 7.9% at higher levels, confirming the assay’s reliable performance across conditions.^19^ Further information on the analytical characterization of the assays can be found in the Supplemental Methods.

### Analytical agreement analysis and diagnostic accuracy (Primary endpoint)

Boxplots, a scatter plot, and a quantile regression were calculated to directly compare both NT-proBNP measurements. The quantile regression with restricted cubic splines was used to assess the relation between NT-proBNP-Elecsys and NT-proBNP-Access assays (knots: 39.95 pg/mL, 330 pg/mL, 1,568 pg/mL, 4,865 pg/mL, and 21,076 pg/mL; function in Supplemental Methods). To further evaluate the analytical agreement between the NT-proBNP-Elecsys and NT-proBNP-Access assays, Bland-Altman plots were generated.^20^ These plots were also created separately for the diagnostic groups, AHF vs no AHF, to assess agreement within each category.

The diagnostic accuracy of NT-proBNP plasma concentrations for diagnosing AHF was the primary endpoint and quantified with the use of the area under the receiver operating characteristics curve (AUC). CIs of AUCs and *P* values for comparison of AUCs were calculated according to the DeLong method.^21^

### Secondary diagnostic endpoints: Performance of guideline-recommended NT-proBNP decision values

Based on data generated with the NT-proBNP-Elecsys assay, current clinical practice guidelines recommend a uniform rule-out decision value (<300 pg/mL) and age-adjusted rule-in decision values for NT-proBNP (450 pg/mL if <50 years, 900 pg/mL if 50-75 years, and 1,800 pg/mL if >75 years) in the early diagnosis of AHF.^1^^,^^2^ This results in 3 possible triage pathways: rule out of AHF, gray-zone, and rule in of AHF.^1^^,^^2^ Using the above-mentioned guideline-recommended decision values, safety of ruling out AHF was quantified by calculating sensitivity, negative predictive value, and the percentage of patients triaged toward rule out, whereas accuracy of ruling in AHF was calculated by specificity, positive predictive value, and the percentage patients triaged toward rule in for both assays (Elecsys and Access).^1^^,^^2^^,^^6^ Comparisons were made using the McNemar test for paired proportions.^22^ Disagreement between assays was considered major if 1 assay indicated rule out whereas the other indicated rule in, as this could have significant clinical consequences. Minor disagreements were categorized as rule-out/rule-in vs gray zone and vice versa.

### Subgroup analyses

Subgroup analyses were predefined in women, older patients, obese patients (body mass index [BMI] of 30 kg/m^2^ or higher), patients with renal dysfunction (estimated glomerular filtration rate >60 mL/min, between 30 and 60 mL/min, and <30 mL/min), and the presence of atrial fibrillation on entry to the ED.^1^^,^^2^^,^^6^^,^^23^, ^24^, ^25^ Based on data from large diagnostic studies, lower clinical decision values are recommended in obese patients to avoid missing mild AHF cases and BMI-adjusted clinical decision values have been derived for NT-proBNP-Elecsys: rule-in, if BMI 30 to 34.9 kg/m^2^: 300 pg/mL if <50 years, 600 pg/mL if 50 to 75 years, and 1,200 pg/mL if >75 years and if BMI 30 to 34.9 kg/m^2^: 225 pg/mL if <50 years, 450 pg/mL if 50 to 75 years, and 900 pg/mL if >75 years; and rule-out, if BMI 30 to 34.9 kg/m^2^ 200 pg/mL and if BMI ≥35 kg/m^2^ 150 pg/mL.^1^^,^^2^^,^^13^^,^^26^ Again, using the above-mentioned recommended decision values, we assessed the performance of both NT-proBNP assays.

### Follow-up and prognostic endpoint

Patients were contacted 90 days, 1 year, and 2 years after discharge by telephone or in written form by trained researchers, who were unaware of the patients' NT-proBNP-Access plasma concentrations during the index hospitalization. In case of a possible relevant medical event, such as death or HF rehospitalization, further information was obtained from the hospital medical records, the general practitioner of the patient, or the national death registry. All-cause death was the primary prognostic endpoint. Time-dependent receiver operating characteristic (ROC) curve and area under time-dependent ROC curves in the presence of censored data were constructed to assess and compare the accuracy of NT-proBNP-Access and NT-proBNP-Elecsys for predicting all-cause mortality within 2 years. A time-dependent ROC curve varies as a function of time and accommodates censored data.^27^ The “timeROC” package in R was used, as it takes into account censored event times with competing risks. Furthermore, Kaplan-Meier curves and log-rank tests were performed.

### General statistical methods

Continuous variables are presented as median (IQR), and categorical variables as numbers and percentages. Comparisons between groups were made by chi-square test, Mann-Whitney *U* test, Kruskal-Wallis test, and Wilcoxon Signed-Rank test, as appropriate. No imputation was performed for missing values. This was an exploratory analysis within a prospective study, and sample size of the overall cohort was not determined specifically for this analysis.^14^^,^^15^ All hypothesis testing was 2-sided and a *P* value <0.05 was regarded as significant. Statistical analyses were carried out by using SPSS/PC Software Package (version 29; SPSS Inc) and R Statistical Software (version 4.3.1; MathSoft), including packages “pROC”, “timeROC”, “rms”, “survival”, “ggplot2”, “tableone”, “haven”, and “tibble”.

## Results

### Patient demographics and characteristics

This analysis included 1,400 patients enrolled between April 11, 2006, and February 27, 2014 (Supplemental Figure 1). The median age was 76 years (IQR: 64-83), with 644 (46%) women and a median BMI of 25.9 kg/m^2^ (IQR: 22.5-30). Of these patients, 744 (53%) were adjudicated as having AHF (Table 1). Among the patients without AHF, the most common causes of acute dyspnea were obstructive pulmonary disease (22%) and pneumonia (15%).

**Table 1:** Baseline Characteristics of the Total Cohort and Stratified by Presence of Acute Heart Failure

	All Patients (N = 1,400)	Non-AHF (n = 656)	AHF (n = 744)	*P* Value^a^
Demographics, median [IQR] or n (%)				
Age, y	76 [64, 83]	69 [56, 78]	80 [71, 85]	<0.001
Body mass index, kg/m^2^	25.9 [22.5, 30.1]	25.6 [21.8, 29.7]	26.1 [23.1, 30.1]	0.019
Female	644 (46)	314 (48)	330 (44)	0.207
Recent history, n (%)				
Chest pain	527 (38)	279 (43)	248 (34)	0.001
Nocturia	423 (49)	172 (42)	251 (56)	<0.001
Weight gain	323 (25)	82 (13)	241 (35)	<0.001
Orthopnea	636 (48)	236 (38)	400 (56)	<0.001
Cough	824 (61)	414 (65)	410 (57)	0.002
Sputum production	564 (41)	285 (45)	279 (39)	0.025
Fever	275 (21)	178 (29)	97 (14)	<0.001
Vital signs at presentation, median [IQR]				
Systolic BP, mm Hg	137 [120, 155]	137 [122, 156]	135 [118, 155]	0.214
Diastolic BP, mm Hg	78 [67, 90]	79 [68, 89]	78 [65, 92]	0.459
Heart rate, beats/min	90 [75, 106]	92 [78, 105]	88 [72, 108]	0.058
Temperature, °C	37.2 [36.8, 37.7]	37.4 [36.9, 37.9]	37.1 [36.6, 37.6]	<0.001
Oxygen saturation, %	96 [93, 98]	96 [93, 99]	96 [93, 98]	0.470
Physical examination at admission, n (%)				
Heart murmur	345 (26)	84 (14)	261 (36)	<0.001
Pulmonary rales	625 (46)	189 (30)	436 (60)	<0.001
Pulmonary wheezing	335 (25)	186 (30)	149 (21)	<0.001
Elevated JVP	331 (26)	43 (7)	288 (42)	<0.001
Positive hepatojugular reflux	117 (9)	21 (4)	96 (14)	<0.001
Edema	592 (43)	151 (24)	441 (60)	<0.001
Ascites	30 (2)	6 (1)	24 (3)	0.006
Chronic comorbidities, n (%)				
COPD/asthma	480 (34)	282 (43)	198 (27)	<0.001
Renal insufficiency	399 (29)	95 (15)	304 (41)	<0.001
Peripheral vascular disease	167 (12)	46 (7)	121 (16)	<0.001
Stroke	160 (11)	54 (8)	106 (14)	0.001
History of pulmonary embolism	120 (9)	65 (10)	55 (7)	0.114
Liver disease	142 (10)	71 (11)	71 (10)	0.482
Active malignancy	62 (5)	14 (3)	48 (7)	0.001
Mental health disorder	326 (23)	158 (24)	168 (23)	0.548
Known heart disease, n (%)				
Chronic HF	456 (33)	67 (10)	389 (52)	<0.001
Hypertensive heart disease	336 (24)	85 (13)	251 (34)	<0.001
Coronary artery disease	495 (35)	128 (20)	367 (49)	<0.001
Percutaneous coronary intervention	205 (15)	57 (9)	148 (20)	<0.001
Coronary bypass	140 (10)	25 (4)	115 (15)	<0.001
Myocardial infarction	273 (20)	67 (10)	206 (28)	<0.001
Valvular replacement	64 (5)	8 (1)	56 (8)	<0.001
History of atrial fibrillation	383 (27)	63 (10)	320 (43)	<0.001
Cardiovascular risk factors, n (%)				
Hypertension	987 (71)	376 (57)	611 (82)	<0.001
Ever smoked	930 (68)	454 (71)	476 (65)	0.037
Dyslipidemia	611 (44)	223 (34)	388 (52)	<0.001
Diabetes mellitus	331 (24)	111 (17)	220 (30)	<0.001
ECG and echocardiographic parameters at presentation, n (%) or median [IQR]				
Sinus rhythm	887 (68)	521 (89)	366 (50)	<0.001
Atrial fibrillation	338 (26)	35 (6)	303 (42)	<0.001
LVEF^b^	53 [40, 60]	60 [55, 63]	45 [32, 60]	<0.001
LVEF <40%	168 (12)	7 (1)	161 (22)	
LVEF 40-49%	98 (7)	16 (2)	82 (11)	
LVEF ≥50%	407 (29)	182 (28)	225 (30)	
AHF phenotypes, n (%)				
ASC and AHF	63 (5)	0 (0)	63 (9)	
Hypertensive AHF	69 (5)	0 (0)	69 (9)	
Isolated right HF	24 (2)	0 (0)	24 (3)	
Worsening or decompensated chronic HF	557 (40)	0 (0)	557 (75)	
Other causes for dyspnea, n (%)				
ACS only	6 (0)	6 (1)	0 (0)	0.028
Arrhythmia only^c^	13 (1)	13 (2)	0 (0)	<0.001
COPD/asthma	309 (22)	227 (35)	82 (11)	<0.001
Pneumonia	215 (15)	138 (21)	77 (10)	<0.001
Carcinoma/metastasis	75 (5)	53 (8)	22 (3)	<0.001
Pulmonary embolism	71 (5)	62 (9)	9 (1)	<0.001
Laboratory values, median [IQR]				
hs-cTnT, *ng/L*	24 [12, 49]	13 [7, 27]	37 [21, 70]	<0.001
Hemoglobin, g/L	133 [118, 146]	139 [126, 150]	128 [113, 141]	<0.001
eGFR^d^, mL/min per 1,73 m^*2*^	67 [44, 88]	83 [61, 99]	53 [35, 75]	<0.001
eGFR >60 mL/min/1.73 m^2^	795 (57)	492 (75)	303 (41)	
eGFR 30–60 mL/min/1.73 m^2^	402 (29)	120 (18)	282 (38)	
eGFR <30 mL/min/1.73 m^2^	181 (13)	34 (5)	147 (20)	
Sodium, mmol/L	138 [135, 141]	138 [135, 140]	139 [136, 141]	<0.001
Potassium, mmol/L	4.1 [3.8, 4.4]	4.0 [3.7, 4.3]	4.1 [3.8, 4.5]	<0.001
CRP, mg/dL	12.6 [3.8, 46.8]	12.9 [3.3, 56.2]	12.3 [4.2, 38.4]	0.534
Chronic Medication, n (%)				
ACEIs/ARBs	714 (51)	244 (37)	470 (64)	<0.001
Beta-blocking agents	639 (46)	181 (28)	458 (62)	<0.001
Diuretics	744 (54)	229 (35)	515 (70)	<0.001

ACEI = angiotensin-converting enzyme inhibitor; ACS = acute coronary syndrome; AHF = acute heart failure; ARB = angiotensin receptor blockers; BP = blood pressure; CRP =C-reactive protein; COPD = chronic obstructive pulmonary disease; ECG = electrocardiogram; eGFR = estimated glomerular filtration rate; HF = heart failure; hs-cTnT = high-sensitivity cardiac troponin T; JVP = jugular venous pressure; LVEF = left ventricular ejection fraction.

a Comparisons were performed using Mann-Whitney *U* test or chi-square test, as appropriate.

b Available in 673 patients.

c Atrial fibrillation, ventricular tachycardia, bradycardia, and atrial ventricular block.

d eGFR was calculated using the Chronic Kidney Disease Epidemiology Collaboration formula.

### Agreement analysis

**Figure 1:**
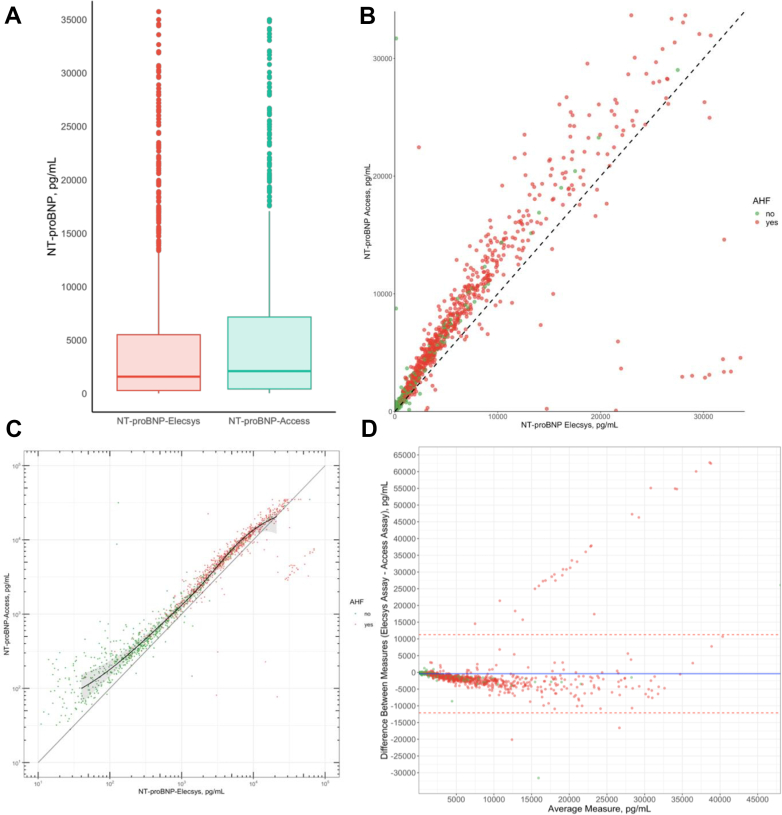
**Direct Comparison of Both NT-proBNP Assays** (A) Boxplots show the distribution of NT-proBNP levels (pg/mL) for the NT-proBNP-Access and NT-proBNP-Elecsys assays in the overall cohort. Bars indicate medians; box bottoms and tops, 25th and 75th percentiles; whiskers, upper and lower adjacent values; dots, outliers. (B) Scatterplot with NT-proBNP-Elecsys on x-axis and NT-proBNP-Access on y-axis. Red dots represent patients with AHF, green, without AHF, black dashed line x = y. (C) Quantile regression with restricted cubic splines using 5 knots. Red dots indicate patients with AHF, green dots, patients without; gray diagonal represents x = y. (D) The x-axis represents the mean of NT-proBNP measurements (Elecsys and Access) for a pertaining patient. The y-axis represents the difference between the NT-proBNP concentrations (Elecsys-Access). The blue line is the mean of the difference between both assays. The red dashed lines are the upper and lower limits of agreement (mean of the difference +/− SD of the difference∗1.96). Patients are stratified according to the final adjudicated diagnosis. AHF = acute heart failure; NT-proBNP = N-terminal pro–B-type natriuretic peptide.

Concentrations of NT-proBNP measured by the Access assay were significantly higher compared to those measured by the Elecsys assay, with a median of 2,087 pg/mL (IQR: 420-7,150 Access) vs 1,568 pg/mL (IQR: 271-5,501 Elecsys; *P* for comparison <0.001). Higher NT-proBNP-Access levels were documented in all examined subgroups (Supplemental Table 2). The distribution of these values is presented in a boxplot, a scatterplot, and a Bland-Altman plot (Figure 1). The quantile regression demonstrated that median NT-proBNP-Access concentrations were significantly higher at lower values, with the difference diminishing at higher concentrations (Figure 1) (with NT-proBNP-Access being 50% higher at NT-proBNP-Elecsys 300 pg/mL, 32% higher at 1,000 pg/mL, 38% higher at 3,000 pg/mL, 35% higher at 10,000 pg/mL, and only 0.3% higher at 20,000 pg/mL). This finding was consistent across both diagnostic groups (AHF vs no AHF) (Supplemental Figure 2). In a small subset of patients with AHF (n = 16), NT-proBNP concentrations measured by the Elecsys assay exceeded 30,000 pg/mL, whereas those measured by the Access assay remained below 5,000 pg/mL. These cases, visible as outliers in the scatterplot and forming a linear distribution in the Bland-Altman plot (Figures 1B and 1D), were characterized by older age, impaired renal function and reduced left ventricular ejection fraction, volume overload (>80% presented with edema and pulmonary rales), and were predominantly admitted for worsening chronic heart failure. Laboratory parameters showed no indication of hemolysis, with hemoglobin levels in the low normal range and normal potassium, bilirubin, and aspartate aminotransferase levels. Detailed characteristics of this subgroup are provided in Supplemental Table 3.

### Primary endpoint: Diagnostic accuracy

**Figure 2:**
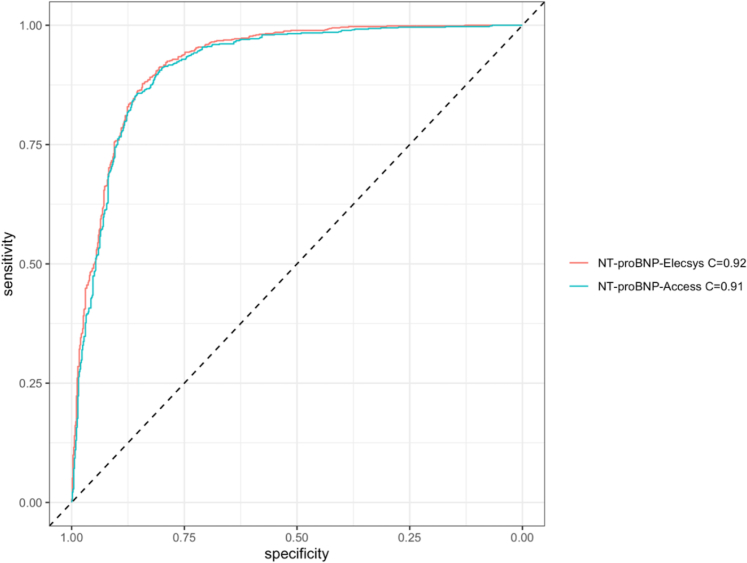
**Diagnostic Accuracies of NT-proBNP Assays in Diagnosing Acute Heart Failure** The area under the receiver operating characteristics curve is 0.914 (95% CI: 0.898-0.930) for the NT-proBNP-Access and 0.922 (95% CI: 0.908-0.937) for the NT-proBNP-Elecsys (DeLong test *P* = 0.006). Abbreviations as in Figure 1.

**Figure 3:**
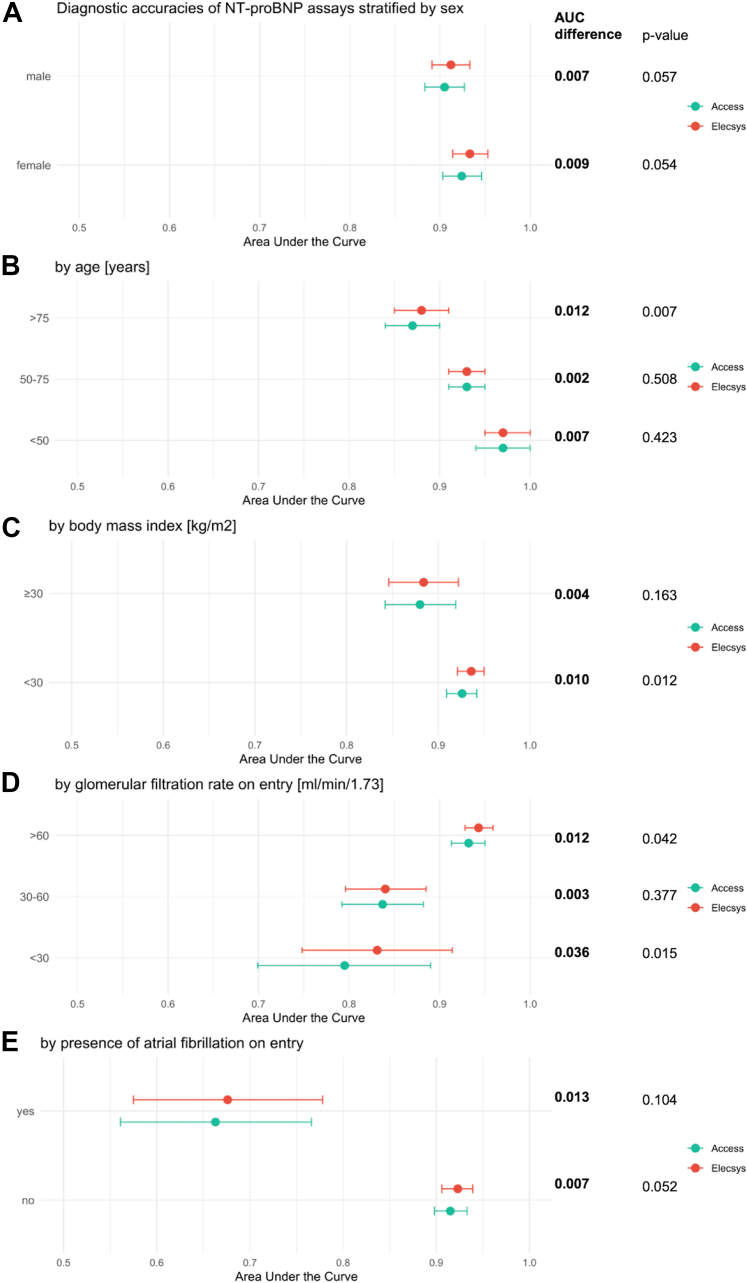
**Performance of Both Assays Across Predefined Subgroups** Clinical subgroups: (A) sex, (B) age (<50, 50-75, >75 years), (C) body mass index (<30, ≥30 kg/m^2^), (D) renal function (eGFR >60, 30-60, <30 mL/min/1.73 m^2^), and (E) atrial fibrillation on admission. *P* value for comparison between the 2 depicted groups calculated using DeLong test, eGFR was calculated using the Chronic Kidney Disease Epidemiology Collaboration formula. AUC = Area under the receiver operating characteristics curve; eGFR = estimated glomerular filtration rate; other abbreviations as in Figure 1.

The diagnostic accuracy of NT-proBNP-Access assay for AHF at ED presentation as quantified using the AUC was very high (0.914; 95% CI: 0.898-0.93), and slightly lower in direct comparison to the NT-proBNP-Elecsys assay (AUC: 0.922; 95% CI: 0.908-0.937; *P* = 0.006) (Figure 2). Comparable diagnostic accuracies between the 2 NT-proBNP assays were confirmed in all predefined subgroup analyses and based on categorical thresholds with minimal AUC differences. No significant interactions between assay type and the subgroups were observed (Figure 3, Supplemental Figure 3, Supplemental Tables 4 and 5).

### Performance of guideline-recommended NT-proBNP decision values

**Figure 4:**
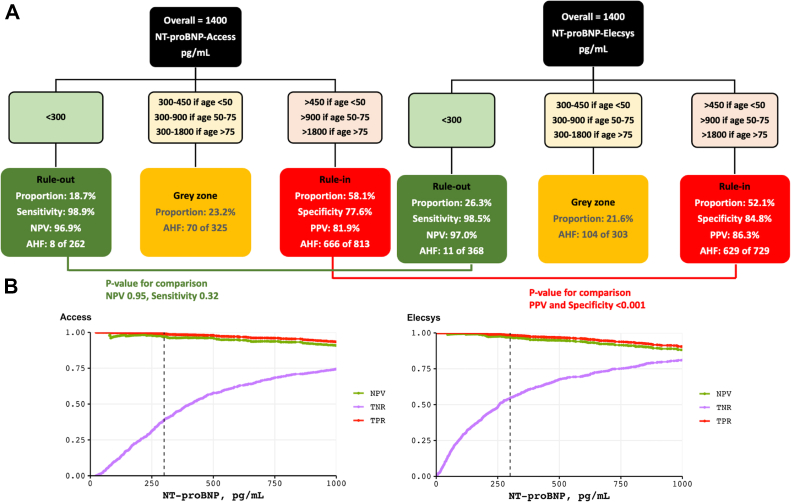
**Triage Pathway to Diagnose Acute Heart Failure** (A) Performance of NT-proBNP-Access and NT-proBNP-Elecsys using the universal NT-proBNP rule out cutoff concentration (<300 pg/mL) and age-dependent rule in cutoff concentrations (>450 pg/mL if < 50 years, >900 pg/mL if 50-75 years, and >1,800 pg/mL if >75 years). (B) Sensitivity (TPR), specificity (TNR) and negative predictive value (NPV) for NT-proBNP-Access and NT-proBNP-Elecsys levels using the universal NT-proBNP rule out cutoff concentration (<300 pg/mL, dashed vertical line). AHF = acute heart failure; NPV = negative predictive value; PPV = positive predictive value; TNR = true negative rate; TPR = true positive rate; other abbreviations as in Figure 1.

**Figure 5:**
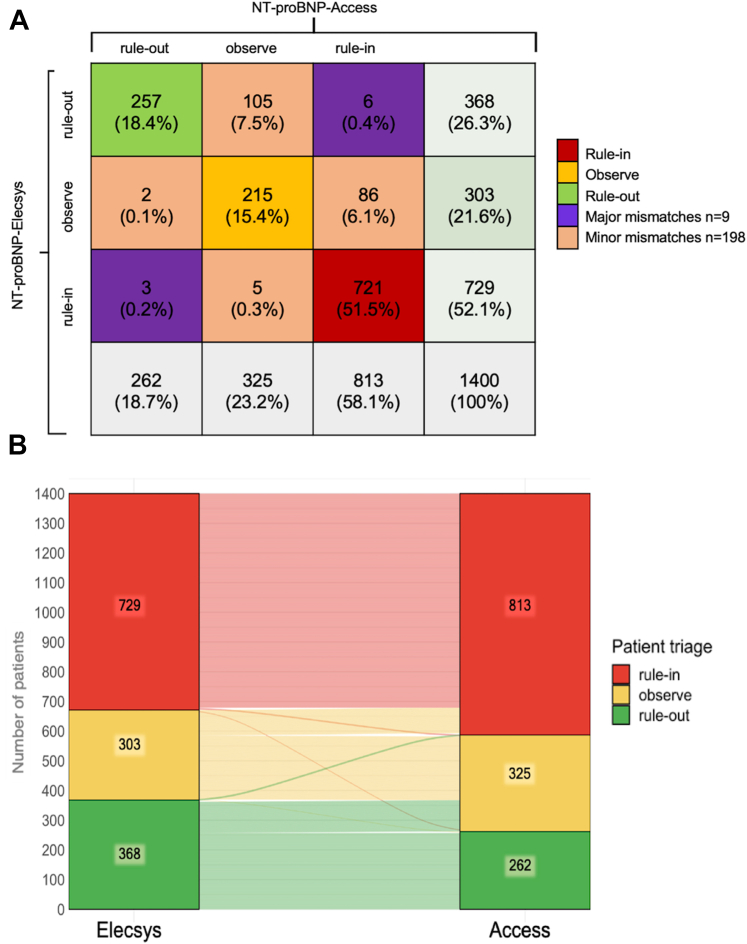
**Diagnostic Concordance Between NT-proBNP-Access and NT-proBNP-Elecsys** (A) A disagreement was considered major (lilac) in those cases where the triage decision for 1 assay was rule out (red) whereas for the other one it was rule in (green). Minor disagreements (nude) were defined as instances where 1 assay indicated rule out whereas the other indicated the gray zone (yellow), or when 1 assay indicated the gray zone whereas the other indicated rule-in. These values represent differences in triage categorization (rule-in, rule-out, or gray zone) between assays and do not correspond to false or true positives. Data are represented as a number (percent). (B) Alluvial plot visualizing the diagnostic concordance for acute heart failure between patient classifications into rule-in (red), observe (yellow), and rule-out (green) using either the NT-proBNP-Elecsys assay (left) or the NT-proBNP-Access assay (right). Numbers in triage feels show the absolute number of patients categorized by each assay. Abbreviations as in Figure 1.

Using guideline-recommended clinical decision values, fewer patients were ruled out (18.7% vs 26.3%) with similar sensitivity (98.9% vs 98.5%; *P* = 0.32), and more patients were ruledin (58.1% vs 52.1%) but with lower specificity (77.6% vs 84.8%; *P* < 0.001) with the NT-proBNP-Access assay vs NT-proBNP-Elecsys leading to a higher false positive rate and in a lower true negative rate (Figure 4; Central Illustration). Similar results were obtained in BMI-adjusted subgroups (Supplemental Figure 4). Diagnostic concordance between assays was high (85.3% in the overall cohort and 84.6% in obese patients). Major mismatches (no AHF vs AHF) were uncommon (0.6% and 1.7% in obese patients, respectively), but minor mismatches (bidirectional gray-zone vs rule-in/rule-out) were relatively frequent (14.1%, eg, gray zone vs no AHF) (Figure 5, Supplemental Figures 5 and 6).

**Central Illustration:**
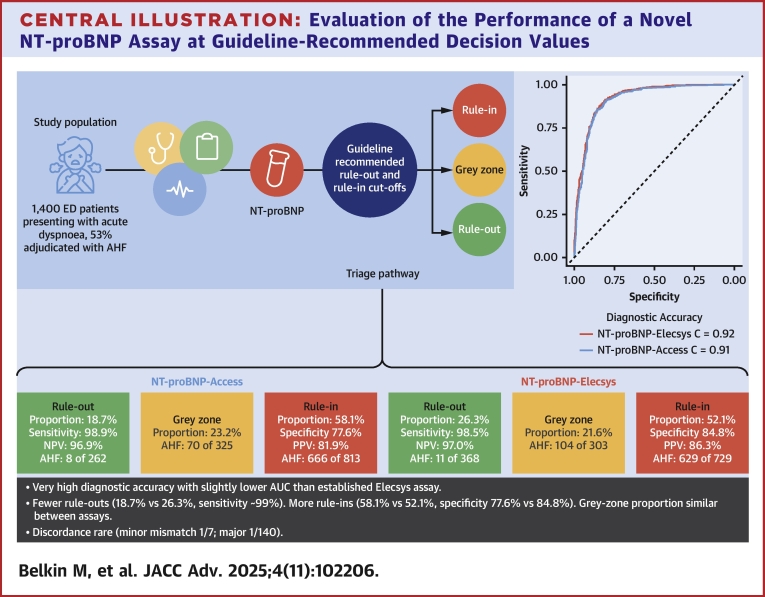
**Evaluation of the Performance of a Novel NT-proBNP Assay at Guideline-Recommended Decision Values** Comparison of the diagnostic accuracy and the performance of NT-proBNP-Access and NT-proBNP-Elecsys using the universal NT-proBNP rule-out cut-off concentration (<300 pg/mL) and age-dependent rule-in cut-off concentrations (>450 pg/mL if <50 years, >900 pg/mL if 50-75 years, and >1,800 pg/mL if >75 years). AHF = acute heart failure; AUC = area under the receiver operating characteristic curve; ED = emergency department; NPV = negative predictive value; NT-proBNP = N-terminal pro–B-type natriuretic peptide; PPV = positive predictive value.

### Prognostic accuracy

Mortality rates during follow-up were substantial, with 22% (314 patients) of the cohort deceased within 1 year and 30% (419 patients) within 2 years. All-cause mortality was significantly higher in patients with a final diagnosis of AHF vs those with other causes of acute dyspnea (Supplemental Figure 7). In the overall cohort, both NT-proBNP-Access and NT-proBNP-Elecsys had moderate-to-high prognostic accuracy for 1-year all-cause mortality (AUC: 0.71; 95% CI: 0.68-0.74 vs AUC: 0.73; 95% CI: 0.70-0.76; *P* = 0.002) and for 2-year all-cause mortality (AUC: 0.71; 95% CI: 0.68-0.74 vs AUC: 0.72; 95% CI: 0.69-0.75; *P* = 0.003). The reliability of these findings within the 2-year follow-up was confirmed using time-dependent AUC (Supplemental Figure 8).

## Discussion

This large multicentre diagnostic study with central adjudication of AHF, to the best of our knowledge, was the first to evaluate the following: 1) the diagnostic accuracy of the novel NT-proBNP-Access assay for AHF at ED presentation in direct comparison with the NT-proBNP-Elecsys; and 2) the performance of guideline-recommended decision values using the Access-NT-proBNP assay in the early diagnosis of AHF. We report 5 major findings:

First, the NT-proBNP-Access assay yielded significantly higher values than the NT-proBNP-Elecsys assay, irrespective of the presence or absence of adjudicated AHF. The relatively large difference between the 2 assays was unexpected, but possibly could have been anticipated given the upper reference limits derived from the specific healthy reference population used for the NT-proBNP-Access assay (age <50 years: upper reference limits [97.5th percentile] 162 pg/mL age 50 to 75 years: upper reference limits of 311 pg/mL; age >75 years: upper reference limits of 457 pg/mL). These were substantially higher than the upper reference limits reported for the NT-proBNP-Elecsys assay in prior presumably healthy reference populations (eg, age 65-74 years: upper reference limits [97.5th percentile] of 879 pg/mL), on which the guideline-recommended <125 pg/mL for the rule out of chronic HF in the nonacute setting is based.^28^^,^^29^ This suggests that recalibration or revised clinical decision values may be required when using NT-proBNP-Access to rule out chronic HF in the nonacute setting. Notably, a small subset of AHF patients had NT-proBNP values measured by the Elecsys assay exceeding 30,000 pg/mL, whereas those from the Access assay remaining below 5,000 pg/mL. This discrepancy formed a *linear pattern* in the Bland-Altman plot, possibly linked to severe renal and cardiac dysfunction. A potential explanation may lie in an altered volume distribution and reduced clearance which lead to accumulation of fragments and might interfere with assay-specific detection of NT-proBNP. Second, the diagnostic accuracy of NT-proBNP-Access assay for AHF as quantified using the AUC was very high (0.914), and only slightly lower in direct comparison to the NT-proBNP-Elecsys assay (AUC: 0.922). Although statistically significant, the difference (0.006) is minimal and clinically irrelevant. This indicates that the highlights that as a diagnostic test, NT-proBNP-Access is as valuable as NT-proBNP-Elecsys. Third, although comparable diagnostic accuracy was confirmed across most predefined subgroups, important variations in AUC among the different subgroups emerged, highlighting lower diagnostic accuracy of NT-proBNP in older patients, patients with obesity, renal dysfunction, and most prominently atrial fibrillation. These findings extend and corroborate prior research.^23^^,^^25^^,^^30^^,^^31^ Fourth, applying guideline-recommended NT-proBNP clinical decision values for AHF for NT-proBNP-Access measurements resulted in fewer patients ruled out (18.7% vs 26.3%) with similar sensitivity (98.9% vs 98.5%), and more patients ruled in (58.1% vs 52.1%) with lower specificity (77.6% vs 84.8%; *P* < 0.001) vs NT-proBNP-Elecsys. Diagnostic concordance between the assays was high (85.3%), with major discordance (no AHF vs AHF) being rare (0.6%), although minor discordance was more common (14.1%, eg, gray zone vs no AHF). From a clinical perspective, especially in settings where treatment decisions are time sensitive, a patient falling into the gray zone with one assay but not the other might experience a delay in treatment initiation or unnecessary admission for further workup. However, the overall proportion of patients remaining in the gray zone was comparable between assays, and the high specificity of NT-proBNP-Access in our study supports its use with the same clinical decision thresholds recommended for NT-proBNP-Elecsys. Alternatively, further research could explore 2 approaches: 1) the NT-proBNP Access assay could be recalibrated to align with established thresholds; and 2) slightly higher novel use-optimized NT-proBNP-Access clinical decision values could be developed and externally validated to increase the proportion of patients eligible for the early rule out of AHF with high safety. To confirm whether the observed differences persist and to clarify the most appropriate strategy additional research using fresh samples could be conducted. Fifth, NT-proBNP-Access showed moderate-to-high prognostic accuracy for both, 1-year (AUC: 0.71) and 2-year all-cause mortality (AUC: 0.71), which was only slightly lower in direct comparison to NT-proBNP-Elecsys. These findings were reliable over the 2-year follow-up period. Although statistically significant, however, the differences (0.02 and 0.01) were small and unlikely to be clinically meaningful.

### Study Limitations

Several limitations should be considered when interpreting these findings. First, this study required written informed consent. Therefore, we cannot comment on the diagnostic accuracy and the performance of guideline-recommended NT-proBNP clinical decision values for AHF using NT-proBNP-Access in critically ill patients unable to provide written informed consent. Second, despite central adjudication of AHF by 2 independent cardiologists/internists using comprehensive clinical information, including cardiac imaging, a small degree of misclassification is possible. Third, although NT-proBNP levels were measured using the Access assay on the same blood samples as the Elecsys assay, the Access measurements were conducted later. Although our analysis used samples that had been stored at −80 °C and the long-term stability of NT-proBNP as an analyte is well established, it is conceivable that the different storage times at −80 °C might have contributed to some of the differences observed, for example, by potential sample degradation.^32^^,^^33^ However, given the fact that the concentrations of NT-proBNP-Access were significantly higher when compared with NT-proBNP-Elecsys, sample degradation is very unlikely. In the small number of patients (n = 16) with substantially lower NT-proBNP measured by Access compared to Elecsys assay, sample degradation cannot be excluded as a contributing factor. However, in these rare cases where sample degradation may have occurred, clinical decision-making was not affected. Nevertheless, additional research using fresh samples may aid to fully understand any performance differences between the 2 assays. Fourth, the potential overestimation of NT-proBNP-Elecsys diagnostic performance must be considered, as it was available for gold standard diagnosis in some patients; however, this concern is mitigated by the fact that 99% of patients were adjudicated based on BNP levels. Fifth, patients with terminal renal dysfunction requiring chronic renal replacement therapy were excluded. Thus, we cannot comment on the diagnostic performance of the novel NT-proBNP-Access assay in these vulnerable patients. Further studies specifically enrolling patients with end-stage renal disease are warranted.

## Conclusions

The NT-proBNP-Access assay demonstrated a very high diagnostic accuracy for AHF. Levels were significantly higher using NT-proBNP-Access vs NT-proBNP-Elecsys, resulting in minor diagnostic discordance in 1 of 7 patients and major discordance in 1 of 140 when using guideline-recommended decision values.

Perspectives **COMPETENCY IN MEDICAL KNOWLEDGE:** NT-proBNP-Access provides very high diagnostic accuracy for AHF in the emergency setting, comparable to NT-proBNP-Elecsys, despite yielding higher absolute concentrations.

**TRANSLATIONAL OUTLOOK:** Minor diagnostic discordance between assays suggests a need for potential recalibration or Access-specific thresholds to optimize clinical utility and minimize uncertainty in time-sensitive care. Notably, those findings should be confirmed using head-to-head measurements in fresh samples.

## Funding support and author disclosures

This study was supported by research grants from the 10.13039/501100001711Swiss National Science Foundation, the 10.13039/501100004362Swiss Heart Foundation, the 10.13039/100008375University of Basel, the 10.13039/100016015University Hospital Basel, 10.13039/100000046Abbott, 10.13039/100007818Alere, Beckman Coulter, BRAHMS, 10.13039/100004337Roche, and 10.13039/100016946Singulex. Dr Wussler reported research grants from the 10.13039/501100001711Swiss National Science Foundation (Grant Reference P500PM_225285), the 10.13039/501100004362Swiss Heart Foundation (Grant Reference FF22112), the 10.13039/100016015University Hospital Basel and the 10.13039/501100005971German Heart Foundation (K22/13) as well as speakers honoraria from PHC outside the submitted work. Dr Lopez-Ayala has received research grants from the 10.13039/501100004362Swiss Heart Foundation (FF20079, FF21103, and FF24149) and speaker’s honoraria from Quidel and 10.13039/100004337Roche, paid to the institution and outside the submitted work. Dr Gualandro reports receiving advisory fees from 10.13039/100004337Roche, outside the submitted work. Dr Kozhuharov reported research grants received from the 10.13039/501100001711Swiss National Science Foundation (grant numbers P400PM-194477 and P5R5PM_210856), the 10.13039/501100000860European Society of Cardiology, the Gottfried und Julia Bangerter-Rhyner Foundation, the Freiwillige Akademische Gesellschaft Basel, and the L. & Th. La Roche Foundation Basel. Dr Mahfoud has been supported by 10.13039/501100001659Deutsche Forschungsgemeinschaft (SFB TRR219, Project-ID 322900939), and 10.13039/501100005971Deutsche Herzstiftung. Saarland University has received scientific support from Ablative Solutions, 10.13039/100004374Medtronic and 10.13039/100015371ReCor Medical; until May 2024, he has received speaker honoraria/consulting fees from Ablative Solutions, Astra-Zeneca, Inari, Medtronic, Merck, 10.13039/100004336Novartis, Philips and ReCor Medical. Dr Hammerer-Lercher reports speaker honoraria from 10.13039/100014386Abbott Diagnostics, Beckman Diagnostics and 10.13039/501100011699Siemens Healthineers. Dr Breidthardt reported research grants from the 10.13039/501100001711Swiss National Science Foundation, the 10.13039/100016015University Hospital Basel, the 10.13039/100017094Department of Internal Medicine, 10.13039/100016015University Hospital Basel, 10.13039/100000046Abbott, and 10.13039/100004337Roche. Dr Mueller reported research grants from the 10.13039/100016015University Hospital Basel, the 10.13039/100008375University of Basel, the 10.13039/501100001711Swiss National Science Foundation, the 10.13039/501100004362Swiss Heart Foundation, the 10.13039/501100013348Innosuisse, 10.13039/100000046Abbott, Astra Zeneca, Beckman Coulter, 10.13039/100001003Boehringer Ingelheim, BRAHMS, Ortho Clinical, Quidel, 10.13039/100004336Novartis, 10.13039/100004337Roche, 10.13039/100004340Siemens, 10.13039/100016946Singulex, SpinChip, and 10.13039/501100018868Sphingotec, as well as speaker/consulting honoraria from 10.13039/100000046Abbott, 10.13039/100000042Amgen, Astra Zeneca, 10.13039/100004326Bayer, 10.13039/100001003Boehringer Ingelheim, 10.13039/100002491BMS, 10.13039/501100022274Daiichi Sankyo, 10.13039/501100016198Idorsia, 10.13039/100004336Novartis, 10.13039/501100004191Novo Nordisk, Osler, 10.13039/100004337Roche, SpinChip, and Sanofi, all paid to the institution. The sponsors had no role in the design and conduct of the study; collection, management, analysis, and interpretation of the data; and preparation, review, or approval of the manuscript. All other authors have reported that they have no relationships relevant to the contents of this paper to disclose.
