# Supplementary material for: A Novel N-Terminal Pro-B-Type Natriuretic Peptide Assay in the Early Diagnosis of Acute Heart Failure
Source: JACC Adv. 2025 Sep 29;4(11):102206. doi: 10.1016/j.jacadv.2025.102206 (PMC12513002; doi:10.1016/j.jacadv.2025.102206)
Supplement: Supplemental Material [file mmc1.docx]

**SUPPLEMENTAL APPENDIX**

**SUPPLEMENTAL METHODS**

**1. Analytical characterization of Access N-terminal pro-B-type natriuretic peptide assay**

The analytical characterization of the Access N-terminal pro-B-type natriuretic peptide (NT-proBNP) assay is conducted on the DxI 9000 Immunoassay Analyzer. Samples for validation studies included human serum and plasma collected in lithium heparin (Li-hep) and ethylenediaminetetraacetic acid (EDTA) tubes from healthy individuals and patients presenting with symptoms indicative of heart failure. Specimens were processed according to standardized preanalytical procedures, including centrifugation to remove residual fibrin and cellular matter. Samples were stored at -30°C to -15°C to maintain analyte stability, with a maximum of three freeze-thaw cycles permitted.

**Assay Principle**

The NT-proBNP-Access assay is a one-step chemiluminescent immunoenzymatic sandwich assay designed to measure NT-proBNP quantitatively in human plasma and serum. The assay uses paramagnetic particles coated with monoclonal anti-NT-proBNP antibodies to capture the NT-proBNP analyte. An alkaline phosphatase-conjugated anti-NT-proBNP antibody binds to a second epitope, forming a two-site complex. Post-incubation, a magnetic field immobilizes the particles, and unbound materials are washed away. The addition of a chemiluminescent substrate generates light proportional to the NT-proBNP concentration, measured with a luminometer. Quantification is automatically determined via a stored calibration curve.

**Analytical Sensitivity and Specificity**

Analytical sensitivity was assessed according to CLSI EP17-A2 guidelines^1^.

- **Limit of Blank (LoB):** Established at 1.1 ng/L using multiple reagent lots over three days with 240 NT-proBNP-free sample points.^2^
- **Limit of Detection (LoD) and Limit of Quantitation (LoQ):** The LoD and LoQ were both established at 4.8 ng/L, with LoQ defined as the NT-proBNP concentration at a 20 % coefficient of variation (CV).^2^

For specificity testing, potential interferences were examined by spiking lithium heparin plasma samples with 74 common pharmaceuticals and endogenous substances, following CLSI EP07-A3 guidelines.^3^ These included medications such as amiodarone, furosemide, and metoprolol, and biological substances like bilirubin, hemoglobin, and lipids. No significant interference (defined as a >10% deviation) was observed at the tested concentrations.^2^

**Imprecision and Reproducibility**

Assay precision was evaluated across seven NT-proBNP concentration levels (38 ng/L to 23,848 ng/L) per CLSI EP05-A3^4^ guidelines. For each concentration, duplicate samples were analyzed across multiple sites and reagent lots over 20 days.

- **Within-run Precision:** CV ranged from 1.4 % to 4.9 %.
- **Between-run and Between-day Precision:** Between-run CVs were maintained under 2.0 % for all tested concentrations, and between-day CVs remained within 1.5 %.^2^
- **Total Reproducibility:** CV ranged from 3.0% at low levels to 7.9 % at higher concentrations, indicating high assay consistency across sites and reagents.^2^

**Linearity**

The assay's linearity was validated across its entire measuring range, 38 ng/L to 25,000 ng/L, using serial dilutions of NT-proBNP standards, analyzed per CLSI EP06-Ed2 guidelines.^5^ Linearity testing confirmed that results were accurate and proportional across the stated range, with deviations from linearity falling within the acceptable limits defined by Clinical and Laboratory Standards Institute (CLSI) guidelines.^6^

**Reference Interval Establishment**

Reference intervals for NT-proBNP were established based on a multicenter study of 675 healthy individuals stratified by age and sex. Subjects with known cardiovascular, renal, or metabolic conditions were excluded to ensure a healthy reference population. Age-specific reference ranges were calculated to account for the observed variation in NT-proBNP levels with age, adhering to CLSI EP28-A3c guidelines. Results indicated significant age dependency in NT-proBNP levels, with elevated upper reference limits (URLs) observed in older age groups. For example:

- Age <50 years: Median NT-proBNP was 48 ng/L, with a URL (97.5th percentile) of 162 ng/L.
- Age 50-75 years: Median NT-proBNP was 94 ng/L, with a URL of 311 ng/L.
- Age >75 years: Median NT-proBNP was 158 ng/L, with a URL of 457 ng/L.

**Sample Type Comparison**

Equivalency between serum, lithium heparin plasma, and EDTA plasma sample types was confirmed according to CLSI EP35 guidelines.^6^ Passing-Bablok regression and Pearson’s correlation were used to compare NT-proBNP levels across these sample types.

**Correlation Analysis:** High correlation coefficients (r > 0.98) were achieved across all comparisons, with slopes ranging between 0.99 and 1.01 and intercepts close to zero, indicating that NT-proBNP measurements were consistent and unaffected by sample type.

**2. Analytical characterization of Elecsys N-terminal pro-B-type natriuretic peptide assay**

The Elecsys NT-proBNP assay is an electrochemiluminescence immunoassay (ECLIA) used for quantitative NT-proBNP measurements in human serum and plasma. The assay operates on a sandwich principle, utilizing two specific monoclonal antibodies against NT-proBNP epitopes. One antibody is biotinylated, and the other is conjugated with a ruthenium complex.^7^

**Assay Principle**

The assay begins with a patient sample incubated with both antibodies to form a sandwich complex with NT-proBNP. Streptavidin-coated microparticles are then added to bind to the biotinylated antibody, anchoring the complex. The reaction mixture is transferred into an electrode-containing cell, where the particles are immobilized by magnetic force, and unbound substances are washed away with ProCell II M solution. A voltage applied to the electrode triggers the ruthenium to emit light, which is detected by a photomultiplier. The light emission is proportional to the NT-proBNP concentration.

**Calibration and Measurement Range**

Results are calibrated using a two-point calibration and a master curve provided by manufacturer. The assay has a measurement range of 5 to 35,000 pg/mL, with dilution extending up to 70,000 pg/mL.

**Analytical Sensitivity**

The assay’s analytical sensitivity parameters, essential for detecting low NT-proBNP levels, are as follows:

- **Limit of Blank (LoB)**: Approximately 3 pg/mL. This is the highest analyte concentration likely to be observed in a sample without the presence of NT-proBNP.
- **Limit of Detection (LoD)**: Around 5 pg/mL, marking the lowest NT-proBNP concentration reliably detected above the LoB.
- **Limit of Quantification (LoQ)**: Approximately 50 pg/mL, representing the lowest NT-proBNP concentration that meets acceptable precision requirements (typically with a CV below 20%).^7^

**Precision and Reproducibility**

The Elecsys NT-proBNP assay maintains high precision, with intra-assay CVs from 1.8% to 2.7% and total imprecision CVs between 2.4% and 3.2%. These values ensure consistent performance across runs and sample conditions, contributing to its reliability as a diagnostic tool for heart failure.

**3. Additional information on quantile regression**

A quantile regression with restricted cubic splines (knots: 39.95 pg/mL, 330 pg/mL, 1568 pg/mL, 4865 pg/mL, 21,076 pg/mL) was performed to assess the relation between NT-proBNP-Elecsys and NT-proBNP-Access assays.

Function of the quantile regression*:* *NT-proBNP-Access = 46.279139+1.3338696* NT-proBNP-Elecsys -1.3722759e-07*pmax(x-39.95,0)^3^+2.0643096e-07*pmax(x-330,0)^3^-8.9915966e-08*pmax(NT-proBNP-Elecsys -1568,0)^3^+2.2095671e-08*pmax(x-4865.1,0)^3^-1.3830738e-09*pmax(NT-proBNP-Elecsys -21075.8,0)^3^ (***Figure 1***).*

**SUPPLEMENTAL REFERENCES**

1. Approved Guideline – Evaluation of Detection Capability for Clinical Laboratory Measurement Procedures, EP17-A2. June 2012. Clinical and Laboratory Standards Institute.

2. Christenson RH, Alahapperuma D, Allen BR, Guidi JL, Headden G, Peacock WF, Winden N, Januzzi JL. A-009 Analytical Characterization of a Novel NT-proBNP Assay on a Central Laboratory Platform. *Clinical Chemistry* 2024;**70**:hvae106.009.

3. Approved Guideline – Interference Testing in Clinical Chemistry, EP07, 3rd Edition. April 2018. Clinical and Laboratory Standards Institute.

4. Approved Guideline – Evaluation of Precision of Quantitative Measurement Procedures, EP05-A3. October 2014. Clinical and Laboratory Standards Institute.

5. Approved Guideline – Evaluation of the Linearity of Quantitative Measurement Procedures, EP06-Ed2. November 2020. Clinical and Laboratory Standards Institute.

6. Approved Guideline – Assessment of Equivalence or Suitability of Specimen Types for Medical Laboratory Measurement Procedures, EP35, 1^st^ Edition. December 2019. Clinical and Laboratory Standards Institute.

7. Elecsys proBNP II Package Insert. Roche Diagnostics, 2024.

**Supplemental Table 1.** STARD Checklist for reporting studies of diagnostic accuracy.

|  | **Section & Topic** | **No** | **Item** | **Reported on page #** |
| --- | --- | --- | --- | --- |
|  |  |  |  |  |
|  | **TITLE OR ABSTRACT** |  |  |  |
|  |  | **1** | Identification as a study of diagnostic accuracy using at least one measure of accuracy (such as sensitivity, specificity, predictive values, or AUC) | 3 |
|  | **ABSTRACT** |  |  |  |
|  |  | **2** | Structured summary of study design, methods, results, and conclusions  (for specific guidance, see STARD for Abstracts) | 3 |
|  | **INTRODUCTION** |  |  |  |
|  |  | **3** | Scientific and clinical background, including the intended use and clinical role of the index test | 5 |
|  |  | **4** | Study objectives and hypotheses | 5 |
|  | **METHODS** |  |  |  |
|  | *Study design* | **5** | Whether data collection was planned before the index test and reference standard were performed (prospective study) or after (retrospective study) | 6 |
|  | *Participants* | **6** | Eligibility criteria | 6 |
|  |  | **7** | On what basis potentially eligible participants were identified (such as symptoms, results from previous tests, inclusion in registry) | 6 |
|  |  | **8** | Where and when potentially eligible participants were identified (setting, location and dates) | 6 |
|  |  | **9** | Whether participants formed a consecutive, random or convenience series | 6 |
|  | *Test methods* | **10a** | Index test, in sufficient detail to allow replication | 7,8 |
|  |  | **10b** | Reference standard, in sufficient detail to allow replication | 7,8 |
|  |  | **11** | Rationale for choosing the reference standard (if alternatives exist) | n.a. |
|  |  | **12a** | Definition of and rationale for test positivity cut-offs or result categories of the index test, distinguishing pre-specified from exploratory | 8 |
|  |  | **12b** | Definition of and rationale for test positivity cut-offs or result categories of the reference standard, distinguishing pre-specified from exploratory | 8 |
|  |  | **13a** | Whether clinical information and reference standard results were available to the performers/readers of the index test | 6,7 |
|  |  | **13b** | Whether clinical information and index test results were available to the assessors of the reference standard | 6,7 |
|  | *Analysis* | **14** | Methods for estimating or comparing measures of diagnostic accuracy | 8,9 |
|  |  | **15** | How indeterminate index test or reference standard results were handled | 8,9 |
|  |  | **16** | How missing data on the index test and reference standard were handled | 10 |
|  |  | **17** | Any analyses of variability in diagnostic accuracy, distinguishing pre-specified from exploratory | n.a. |
|  |  | **18** | Intended sample size and how it was determined | n.a. |
|  | **RESULTS** |  |  |  |
|  | *Participants* | **19** | Flow of participants, using a diagram | Suppl. Figure 1 |
|  |  | **20** | Baseline demographic and clinical characteristics of participants | Table 1 |
|  |  | **21a** | Distribution of severity of disease in those with the target condition | 11 |
|  |  | **21b** | Distribution of alternative diagnoses in those without the target condition | 11 |
|  |  | **22** | Time interval and any clinical interventions between index test and reference standard | n.a. |
|  | *Test results* | **23** | Cross tabulation of the index test results (or their distribution) by the results of the reference standard | n.a. |
|  |  | **24** | Estimates of diagnostic accuracy and their precision (such as 95% confidence intervals) | 11 |
|  |  | **25** | Any adverse events from performing the index test or the reference standard | n.a. |
|  | **DISCUSSION** |  |  |  |
|  |  | **26** | Study limitations, including sources of potential bias, statistical uncertainty, and generalisability | 15 |
|  |  | **27** | Implications for practice, including the intended use and clinical role of the index test | 13-14 |
|  | **OTHER INFORMATION** |  |  |  |
|  |  | **28** | Registration number and name of registry | Title page |
|  |  | **29** | Where the full study protocol can be accessed | n.a. |
|  |  | **30** | Sources of funding and other support; role of funders | 16 |
|  |  |  |  |  |

AUC: area under the curve, n.a.: not applicable, STARD: The Standards for Reporting of Diagnostic Accuracy Group.

**Supplemental Table 1B.** STROBE Statement. Checklist of items that should be included in reports of cohort studies***.***

|  | Item No | Recommendation | Page No |
| --- | --- | --- | --- |
| **Title and abstract** | 1 | (*a*) Indicate the study’s design with a commonly used term in the title or the abstract | 1 |
|  |  | (*b*) Provide in the abstract an informative and balanced summary of what was done and what was found | 3-4 |
| Introduction | | | |
| Background/rationale | 2 | Explain the scientific background and rationale for the investigation being reported | 5 |
| Objectives | 3 | State specific objectives, including any prespecified hypotheses | 5 |
| Methods | | | |
| Study design | 4 | Present key elements of study design early in the paper | 6 |
| Setting | 5 | Describe the setting, locations, and relevant dates, including periods of recruitment, exposure, follow-up, and data collection | 6-7 |
| Participants | 6 | (*a*) Give the eligibility criteria, and the sources and methods of selection of participants. Describe methods of follow-up | 6-7 |
|  |  | (*b*) For matched studies, give matching criteria and number of exposed and unexposed |  |
| Variables | 7 | Clearly define all outcomes, exposures, predictors, potential confounders, and effect modifiers. Give diagnostic criteria, if applicable | 8-9 |
| Data sources/ measurement | 8* | For each variable of interest, give sources of data and details of methods of assessment (measurement). Describe comparability of assessment methods if there is more than one group | 9-10 |
| Bias | 9 | Describe any efforts to address potential sources of bias | 6-7 |
| Study size | 10 | Explain how the study size was arrived at |  |
| Quantitative variables | 11 | Explain how quantitative variables were handled in the analyses. If applicable, describe which groupings were chosen and why | 9-10 |
| Statistical methods | 12 | (*a*) Describe all statistical methods, including those used to control for confounding | 9-10 |
|  |  | (*b*) Describe any methods used to examine subgroups and interactions | 9-10 |
|  |  | (*c*) Explain how missing data were addressed | 9 |
|  |  | (*d*) If applicable, explain how loss to follow-up was addressed |  |
|  |  | (*e*) Describe any sensitivity analyses |  |
| Results | | |  |
| Participants | 13* | (a) Report numbers of individuals at each stage of study—e.g. numbers potentially eligible, examined for eligibility, confirmed eligible, included in the study, completing follow-up, and analysed | 11 |
|  |  | (b) Give reasons for non-participation at each stage |  |
|  |  | (c) Consider use of a flow diagram | supplement |
| Descriptive data | 14* | (a) Give characteristics of study participants (eg demographic, clinical, social) and information on exposures and potential confounders | 15 |
|  |  | (b) Indicate number of participants with missing data for each variable of interest |  |
|  |  | (c) Summarise follow-up time (eg, average and total amount) | 14 |
| Outcome data | 15* | Report numbers of outcome events or summary measures over time | 13-14 |
| Main results | 16 | (a) Give unadjusted estimates and, if applicable, confounder-adjusted estimates and their precision (eg, 95% confidence interval). Make clear which confounders were adjusted for and why they were included | 11-14 |
|  |  | (b) Report category boundaries when continuous variables were categorized | 11-14 |
|  |  | (c) If relevant, consider translating estimates of relative risk into absolute risk for a meaningful time period |  |
| Other analyses | 17 | Report other analyses done—eg analyses of subgroups and interactions, and sensitivity analyses |  |
| Discussion | | | |
| Key results | 18 | Summarise key results with reference to study objectives | 15 |
| Limitations | 19 | Discuss limitations of the study, taking into account sources of potential bias or imprecision. Discuss both direction and magnitude of any potential bias | 18 |
| Interpretation | 20 | Give a cautious overall interpretation of results considering objectives, limitations, multiplicity of analyses, results from similar studies, and other relevant evidence | 15-17 |
| Generalisability | 21 | Discuss the generalisability (external validity) of the study results | 17 |
| Other information | | | |
| Funding | 22 | Give the source of funding and the role of the funders for the present study and, if applicable, for the original study on which the present article is based | 27 |

*Give information separately for exposed and unexposed groups.

**Supplemental Table 2.** Direct comparison of NT-proBNP measured by the Access assay and the Elecsys assay in predefined subgroups.

|  | All patients  *(n=1400)* | Non-AHF  *(n=656)* | AHF  *(n=744)* |
| --- | --- | --- | --- |
| NT-proBNP Access,  *pg/ml* | 2087  [421, 7148] | 405  [204, 1032] | 6111  [2941, 11357] |
| NT-proBNP Elecsys,  *pg/ml* | 1568.0  [272, 5490] | 256  [91, 731] | 4607  [2100, 9301] |
| Age <50 years | **Overall**  *(n=132)* | **Non-AHF**  *(n=113)* | **AHF**  *(n=19)* |
| NT-proBNP Access,  *pg/ml* | 100  [186, 629] | 153  [90, 284] | 4254  [2267, 7215] |
| NT-proBNP Elecsys,  *pg/ml* | 26  [61, 298] | 52  [21, 120] | 2763  [1391, 5233] |
| Age 50-75 years | **Overall**  *(n=541)* | **Non-AHF**  *(n=311)* | **AHF**  *(n=230)* |
| NT-proBNP Access,  *pg/ml* | 316  [991, 4720] | 371  [190, 776] | 4738  [2193, 8805] |
| NT-proBNP Elecsys,  *pg/ml* | 190  [703, 3499] | 236  [90, 493] | 3491  [1666, 7189] |
| Age >75 years | **Overall**  *(n=727)* | **Non-AHF**  *(n=232)* | **AHF**  *(n=495)* |
| NT-proBNP Access,  *pg/ml* | 1158  [4420, 9867] | 577  [248, 1361] | 5481  [2339, 10624] |
| NT-proBNP Elecsys,  *pg/ml* | 886  [3182, 8097] | 741  [367, 1903] | 6984  [3262, 12488] |

| Female | Overall  *(n=644)* | Non-AHF  *(n=314)* | AHF  *(n=330)* |
| --- | --- | --- | --- |
| NT-proBNP Access,  *pg/ml* | 1663  [390, 6958] | 393  [229, 938] | 5896  [2645, 11158] |
| NT-proBNP Elecsys,  *pg/ml* | 1238  [252, 5081] | 251  [103, 627] | 4504  [1971, 9097] |
| Male | **Overall**  *(n=756)* | **Non-AHF**  *(n=342)* | **AHF**  *(n=414)* |
| NT-proBNP Access,  *pg/ml* | 2537  [460, 7354] | 419  [169, 1121] | 6234  [3065, 11727] |
| NT-proBNP Elecsys,  *pg/ml* | 1853  312, 5852] | 272  [76, 830] | 4622  [2124, 9529] |

| BMI <30 kg/m2 | Overall  *(n=1039)* | Non-AHF  *(n=492)* | AHF  *(n=547)* |
| --- | --- | --- | --- |
| NT-proBNP Access,  *pg/ml* | 2343  [420, 7710] | 400  [200, 1084] | 6895  [3285, 12647] |
| NT-proBNP Elecsys,  *pg/ml* | 1726  [272, 6225] | 257  [96, 781] | 5507  [2339, 10574] |
| BMI >= 30 kg/m2 | **Overall**  *(n=350)* | **Non-AHF**  *(n=160)* | **AHF**  *(n=190)* |
| NT-proBNP Access,  *pg/ml* | 1513  [412, 5363] | 410  [204, 820] | 4332  [1741, 7716] |
| NT-proBNP Elecsys,  *pg/ml* | 1100  [251, 3834] | 243  [76, 646] | 3164  [1282, 6072] |

| eGFR <30 ml/min | Overall  *(n=181)* | Non-AHF  *(n=34)* | AHF  *(n=147)* |
| --- | --- | --- | --- |
| NT-proBNP Access,  *pg/ml* | 8537  [4430, 18631] | 2800  [583, 7916] | 10228  [5987, 19272] |
| NT-proBNP Elecsys,  *pg/ml* | 7741  [4084, 19887] | 2070  [453, 5836] | 10316  [5158, 21687] |
| eGFR 30-60 ml/min | **Overall**  *(n=402)* | **Non-AHF**  *(n=120)* | **AHF**  *(n=282)* |
| NT-proBNP Access,  *pg/ml* | 5088  [1408, 10115] | 938  [402, 2535] | 6878  [3098, 11715] |
| NT-proBNP Elecsys,  *pg/ml* | 3495  [1077, 7794] | 707  [257, 2027] | 5275  [2280, 9063] |
| eGFR >60 ml/min | **Overall**  *(n=795)* | **Non-AHF**  *(n=492)* | **AHF**  *(n=303)* |
| NT-proBNP Access,  *pg/ml* | 738  [261, 3137] | 336  [167, 692] | 4191  [2030, 7408] |
| NT-proBNP Elecsys,  *pg/ml* | 505  [125, 2120] | 192  [70, 469] | 2889  [1468, 5853] |

| Atrial fibrillation  yes | Overall  *(n=338)* | Non-AHF  *(n=35)* | AHF  *(n=303)* |
| --- | --- | --- | --- |
| NT-proBNP Access,  *pg/ml* | 5853  [3113, 10571] | 3623  [1145, 7226] | 6113  [3285, 10837] |
| NT-proBNP Elecsys,  *pg/ml* | 4293  [2207, 8631] | 2451  [953, 5153] | 4449  [2430, 8842 |
| Atrial fibrillation  no | **Overall**  *(n=972)* | **Non-AHF**  *(n=548)* | **AHF**  *(n=424)* |
| NT-proBNP Access,  *pg/ml* | 1125  [334, 5882] | 392  [207, 929] | 6064  [2519, 11887] |
| NT-proBNP Elecsys,  *pg/ml* | 849  [204, 4350] | 255  [101, 666] | 4676  [1797, 9887] |

AHF: Acute heart failure. eGFR: estimated glomerular filtration rate. NT-proBNP: N-terminal pro–B-type natriuretic peptide.

Atrial fibrillation on entry to emergency department, eGFR was calculated using the Chronic Kidney Disease Epidemiology Collaboration formula.

**Supplemental Table 3.** Baseline characteristics of patients with NT-proBNP-Elecsys >30,000 pg/mL and NT-proBNP-Access <5,000 pg/mL.

|  | All patients *(n=16)* |
| --- | --- |
| Demographics, *median (IQR)* |  |
| Age, years | 82.5 [77.0, 85.0] |
| Body mass index, *kg/m^2^* | 24.4 [21.2, 27.0] |
| Female sex | 7 (44) |
| All-cause mortality within 720 days, *n (%)* | 12 (75) |
| Recent history, *n (%)* |  |
| Chest pain | 7 (44) |
| Nocturia | 6 (67) |
| Weight gain | 6 (40) |
| Orthopnea | 14 (88) |
| Cough | 6 (38) |
| Sputum production | 2 (12) |
| Fever | 1 (6) |
| Vital signs at admission, *median (IQR)* |  |
| Systolic BP, *mmHg* | 116.0 [104.8, 130.2] |
| Diastolic BP, *mmHg* | 79.5 [68.8, 87.0] |
| Heart rate, *beats/min* | 75.5 [67.2, 90.5] |
| Temperature, *°C* | 36.8 [36.0, 37.4] |
| Oxygen saturation, *%* | 97.0 [96.0, 99.0] |
| Physical examination at admission, *median (IQR)* |  |
| Heart murmur | 9 (56) |
| Pulmonary rales | 12 (80) |
| Pulmonary wheezing | 15 (100) |
| Elevated JVP | 8 (53) |
| Positive hepatojugular reflux | 5 (31) |
| Edema | 13 (81) |
| Ascites | 1 (6) |
| Chronic comorbidities, *n (%)* |  |
| COPD/asthma | 2 (12) |
| Renal insufficiency | 12 (75) |
| Peripheral vascular disease | 5 (31) |
| Stroke | 3 (19) |
| Liver disease | 1 (6) |
| Active malignancy | 1 (6) |
| Structural heart disease, *n (%)* |  |
| Chronic heart failure | 12 (75) |
| Hypertensive heart disease | 6 (38) |
| Coronary artery disease | 15 (94) |
| Percutaneous coronary intervention | 4 (25) |
| Coronary bypass | 3 (19) |
| Myocardial infarction | 8 (50) |
| Valvular replacement | 2 (12) |
| History of atrial fibrillation | 7 (44) |
| Cardiovascular risk factors, *n (%)* |  |
| Hypertension | 14 (88) |
| Ever smoked | 10 (62) |
| Dyslipidemia | 11 (69) |
| Diabetes mellitus | 6 (38) |
| ECG and echocardiographic parameters at admission, *n (%)* |  |
| Sinus rhythm | 10 (62) |
| Atrial fibrillation | 6 (38) |
| LVEF^2^ | 20.0 [17.5, 35.0] |
| Heart failure phenotypes, *n (%)* |  |
| ACS and AHF | 1 (6) |
| Worsening or decompensated chronic HF | 14 (88) |
| Cardiogenic shock | 1 (6) |
| Median (IQR) laboratory values |  |
| NT-proBNP-Access, *pg/mL* | 3993.5 [3384.2, 4456.2] |
| NT-proBNP-Elecsys, *pg/mL* | 35378.0 [32491.5, 40548.0] |
| \| hs-cTnT, *ng/L* \| 61.4 (201.0) \| 24.8 (41.2) \| 93.8 (269.2) \| <0.001 \| \| --- \| --- \| --- \| --- \| --- \| | 104.5 [60.5, 134.0] |
| Hemoglobin, *g/L* | 119.5 [106.2, 127.0] |
| eGFR^2^, *mL/min per 1,73 m^2^* | 17.2 [12.1, 27.0] |
| Sodium, *mmol/L* | 140.0 [137.8, 141.2] |
| Potassium, *mmol/L* | 4.2 [3.8, 4.9] |
| CRP, *mg/dL* | 24.2 [11.2, 59.3] |
| Bilirubin, *µmol/L* | 15.5 [10.0, 21.0] |
| Aspartate aminotransferase, *U/L* | 27 [21, 41] |
| Medication on admission, n (%) |  |
| ACEIs/ ARBs | 13 (81) |
| Beta-blocking agents | 14 (88) |
| Diuretics | 14 (88) |

ACEI: Angiotensin-converting enzyme inhibitor. ACS: Acute coronary syndrome. AHF: Acute heart failure. ARB: Angiotensin receptor blockers. BP: Blood pressure. CRP: C-reactive protein. COPD: Chronic obstructive pulmonary disease. eGFR: Estimated glomerular filtration rate. HF: Heart failure. hs-cTnT: high-sensitivity cardiac troponin T. IQR: Interquartile range. JVP: Jugular venous pressure. LVEF: left ventricular ejection fraction. NT-proBNP: N-terminal pro–B-type natriuretic peptide.

* Comparisons were done using Mann–Whitney U test or x2 test, as appropriate.

^1^ Atrial fibrillation, ventricular tachycardia, bradycardia, atrial ventricular block. ^2^ eGFR was calculated using the Chronic Kidney Disease Epidemiology Collaboration formula.

^2^ available in 11 patients

**Supplemental Table 4A.** Comparison of diagnostic accuracies between NT-proBNP-Access and NT-proBNP-Elecsys assay in predefined subgroups.

| **Patient stratification** | **Predictor** | **AUC [95% CI]** | **AUC difference** | **p-value** |  |
| --- | --- | --- | --- | --- | --- |
| Age <50 years | NT-proBNP Access, *pg/ml* | 0.97 [0.94, 1.0] | 0.007 | 0.432 |  |
|  | NT-proBNP Elecsys, *pg/ml* | 0.97 [0.95, 1.0] |  |  |  |
|  | NT-proBNP Access, *pg/ml* | 0.93 [0.91, 0.95] | 0.002 | 0.508 |  |
| Age 50-75 years | NT-proBNP Elecsys, *pg/ml* | 0.93 [0.91, 0.95] |  |  |  |
| Age >75 years | NT-proBNP Access, *pg/ml* | 0.87 [0.84, 0.90] | 0.012 | 0.007 |  |
|  | NT-proBNP Elecsys, *pg/ml* | 0.88 [0.85, 0.91] |  |  |  |
|  |  |  |  |  |  |
| Female | NT-proBNP Access, *pg/ml* | 0.92 [0.90, 0.95] | 0.009 | 0.054 |  |
|  | NT-proBNP Elecsys, *pg/ml* | 0.93 [0.91, 0.95] |  |  |  |
| Male | NT-proBNP Access, *pg/ml* | 0.91 [0.88, 0.93] | 0.007 | 0.054 |  |
|  | NT-proBNP Elecsys, *pg/ml* | 0.91 [0.89, 0.93] |  |  |  |
|  |  |  |  |  |  |
| BMI <30 kg/m2 | NT-proBNP Access, *pg/ml* | 0.93 [0.91, 0.94] | 0.010 | 0.012 |  |
|  | NT-proBNP Elecsys, *pg/ml* | 0.94 [0.92, 0.95] |  |  |  |
| BMI ≥30 kg/m2 | NT-proBNP Access, *pg/ml* | 0.88 [0.84, 0.92] | 0.004 | 0.163 |  |
|  | NT-proBNP Elecsys, *pg/ml* | 0.88 [0.85, 0.92] |  |  |  |
|  |  |  |  |  |  |
| eGFR >60 ml/min | NT-proBNP Access, *pg/ml* | 0.93 [0.91, 0.95] | 0.012 | 0.042 |  |
|  | NT-proBNP Elecsys, *pg/ml* | 0.94 [0.93, 0.96] |  |  |  |
| eGFR 30-60 ml/min | NT-proBNP Access, *pg/ml* | 0.84 [0.79, 0.88] | 0.003 | 0.377 |  |
|  | NT-proBNP Elecsys, *pg/ml* | 0.84 [0.80, 0.89] |  |  |  |
| eGFR <30 ml/min | NT-proBNP Access, *pg/ml* | 0.80 [0.70, 0.89] | 0.036 | 0.015 |  |
|  | NT-proBNP Elecsys, *pg/ml* | 0.83 [0.75, 0.92] |  |  |  |
|  |  |  |  |  |  |
| Atrial fibrillation  yes | NT-proBNP Access, *pg/ml* | 0.66 [0.56, 0.78 | 0.013 | 0.104 |  |
|  | NT-proBNP Elecsys, *pg/ml* | 0.68 [0.57, 0.78] |  |  |  |
| no | NT-proBNP Access, *pg/ml* | 0.92 [0.90, 0.93] | 0.007 | 0.052 |  |
|  | NT-proBNP Elecsys, *pg/ml* | 0.92 [0.91, 0.94] |  |  |  |

**Supplemental Table 4B.** Logistic regression interaction analysis.

| **Factor** | **χ²** | **p-value** |
| --- | --- | --- |
| Assay | 0.0 | 1.00 |
| eGFR category | 326.3 | <0.0001 |
| Assay × eGFR category (interaction) | 0.0 | 1.00 |
| Assay ×age category (interaction) | 0.0 | 1.00 |
| Assay × BMI category (interaction) | 0.0 | 1.00 |
| Assay × sex (interaction) | 0.0 | 1.00 |
| Assay × AF (interaction) | 0.0 | 1.00 |

AHF: acute heart failure, AUC: area under the curve, BMI: body mass index, CI: confidence interval, Dx: other causes for acute dyspnea, eGFR: estimated glomerular filtration rate, NT-proBNP: N-terminal pro-B-type natriuretic peptide.

* p-value for comparison between the two depicted groups, eGFR was calculated using the Chronic Kidney Disease Epidemiology Collaboration formula.

|  | **Patient stratification** | **Predictor** | **AUC [95% CI]** | **p-value** |
| --- | --- | --- | --- | --- |
| Overall cohort | Rule-out | NT-proBNP Access, *pg/ml* | 0.69 [0.67, 0.71] | <0.001 |
|  |  | NT-proBNP Elecsys, *pg/ml* | 0.77 [0.75, 0.78] |  |
|  | 3-level classification | NT-proBNP Access, *pg/ml* | 0.85 [0.83, 0.87] | <0.001 |
|  |  | NT-proBNP Elecsys, *pg/ml* | 0.88 [0.87, 0.90] |  |
|  | Rule-in | NT-proBNP Access, *pg/ml* | 0.84 [0.82, 0.86] | 0.117 |
|  |  | NT-proBNP Elecsys, *pg/ml* | 0.85 [0.83, 0.87] |  |

**Supplemental Table 5.** Comparison of diagnostic accuracies between NT-proBNP-Access and NT-proBNP-Elecsys assay based on categorical thresholds (rule-in only with age-adjusted thresholds, rule-out only <300 pg/mL and combined 3-level classification (rule-out, grey zone, rule-in).

|  | **Patient stratification** | **Predictor** | **AUC [95% CI]** | **p-value** |
| --- | --- | --- | --- | --- |
| Female | Rule-out | NT-proBNP Access, *pg/ml* | 0.69 [0.67, 0.72] | <0.001 |
|  |  | NT-proBNP Elecsys, *pg/ml* | 0.78 [0.75, 0.81] |  |
|  | 3-level classification | NT-proBNP Access, *pg/ml* | 0.87 [0.85, 0.90] | 0.002 |
|  |  | NT-proBNP Elecsys, *pg/ml* | 0.90 [0.88, 0.92] |  |
|  | Rule-in | NT-proBNP Access, *pg/ml* | 0.85 [0.82, 0.88] | 0.776 |
|  |  | NT-proBNP Elecsys, *pg/ml* | 0.86 [0.83, 0.88] |  |

|  | **Patient stratification** | **Predictor** | **AUC [95% CI]** | **p-value** |
| --- | --- | --- | --- | --- |
| Male | Rule-out | NT-proBNP Access, *pg/ml* | 0.68 [0.66, 0.71] | <0.001 |
|  |  | NT-proBNP Elecsys, *pg/ml* | 0.75 [0.73, 0.78] |  |
|  | 3-level classification | NT-proBNP Access, *pg/ml* | 0.83 [0.81, 0.86] | <0.001 |
|  |  | NT-proBNP Elecsys, *pg/ml* | 0.87 [0.84, 0.89] |  |
|  | Rule-in | NT-proBNP Access, *pg/ml* | 0.82 [0.79, 0.85] | 0.059 |
|  |  | NT-proBNP Elecsys, *pg/ml* | 0.84 [0.81, 0.86] |  |
|  | **Patient stratification** | **Predictor** | **AUC [95% CI]** | **p-value** |
| eGFR >60 ml/min | Rule-out | NT-proBNP Access, *pg/ml* | 0.67 [0.70, 0.71] | <0.001 |
|  |  | NT-proBNP Elecsys, *pg/ml* | 0.77 [0.75, 0.78] |  |
|  | 3-level classification | NT-proBNP Access, *pg/ml* | 0.85 [0.83, 0.87] | <0.001 |
|  |  | NT-proBNP Elecsys, *pg/ml* | 0.88 [0.87, 0.90] |  |
|  | Rule-in | NT-proBNP Access, *pg/ml* | 0.84 [0.82, 0.86] | 0.117 |
|  |  | NT-proBNP Elecsys, *pg/ml* | 0.85 [0.83, 0.87] |  |

|  | **Patient stratification** | **Predictor** | **AUC [95% CI]** | **p-value** |
| --- | --- | --- | --- | --- |
| eGFR 30-60 ml/min | Rule-out | NT-proBNP Access, *pg/ml* | 0.57 [0.54, 0.61] | <0.001 |
|  |  | NT-proBNP Elecsys, *pg/ml* | 0.63 [0.59, 0.67] |  |
|  | 3-level classification | NT-proBNP Access, *pg/ml* | 0.75 [0.70, 0.80] | 0.022 |
|  |  | NT-proBNP Elecsys, *pg/ml* | 0.78 [0.74, 0.83] |  |
|  | Rule-in | NT-proBNP Access, *pg/ml* | 0.75 [0.70, 0.79] | 0.165 |
|  |  | NT-proBNP Elecsys, *pg/ml* | 0.77 [0.72, 0.81] |  |
|  | **Patient stratification** | **Predictor** | **AUC [95% CI]** | **p-value** |
| eGFR <30 ml/min | Rule-out | NT-proBNP Access, *pg/ml* | 0.56 [0.50, 0.61] | 1.000 |
|  |  | NT-proBNP Elecsys, *pg/ml* | 0.56 [0.50, 0.61] |  |
|  | 3-level classification | NT-proBNP Access, *pg/ml* | 0.69 [0.60, 0.77] | 0.074 |
|  |  | NT-proBNP Elecsys, *pg/ml* | 0.73 [0.64, 0.82] |  |
|  | Rule-in | NT-proBNP Access, *pg/ml* | 0.68 [0.60, 0.77] | 0.074 |
|  |  | NT-proBNP Elecsys, *pg/ml* | 0.73 [0.64, 0.81] |  |

|  | **Patient stratification** | **Predictor** | **AUC [95% CI]** | **p-value** |
| --- | --- | --- | --- | --- |
| Atrial fibrillation | Rule-out | NT-proBNP Access, *pg/ml* | 0.51 [0.48, 0.54] | 0.112 |
|  |  | NT-proBNP Elecsys, *pg/ml* | 0.54 [0.50, 0.59] |  |
|  | 3-level classification | NT-proBNP Access, *pg/ml* | 0.58 [0.51, 0.65] | 0.394 |
|  |  | NT-proBNP Elecsys, *pg/ml* | 0.60 [0.52, 0.69] |  |
|  | Rule-in | NT-proBNP Access, *pg/ml* | 0.58 [0.51, 0.65] | 0.515 |
|  |  | NT-proBNP Elecsys, *pg/ml* | 0.60 [0.52, 0.68] |  |
|  | **Patient stratification** | **Predictor** | **AUC [95% CI]** | **p-value** |
| No atrial fibrillation | Rule-out | NT-proBNP Access, *pg/ml* | 0.69 [0.67, 0.71] | <0.001 |
|  |  | NT-proBNP Elecsys, *pg/ml* | 0.76 [0.74, 0.78] |  |
|  | 3-level classification | NT-proBNP Access, *pg/ml* | 0.86 [0.84, 0.88] | <0.001 |
|  |  | NT-proBNP Elecsys, *pg/ml* | 0.89 [0.87, 0.91] |  |
|  | Rule-in | NT-proBNP Access, *pg/ml* | 0.84 [0.82, 0.86] | 0.128 |
|  |  | NT-proBNP Elecsys, *pg/ml* | 0.85 [0.83, 0.87] |  |

AUC: area under the curve, CI: confidence interval, NT-proBNP: N-terminal pro-B-type natriuretic peptide.

* p-value for comparison between the two depicted groups.

**Supplemental Figure 1.** Patient flow.


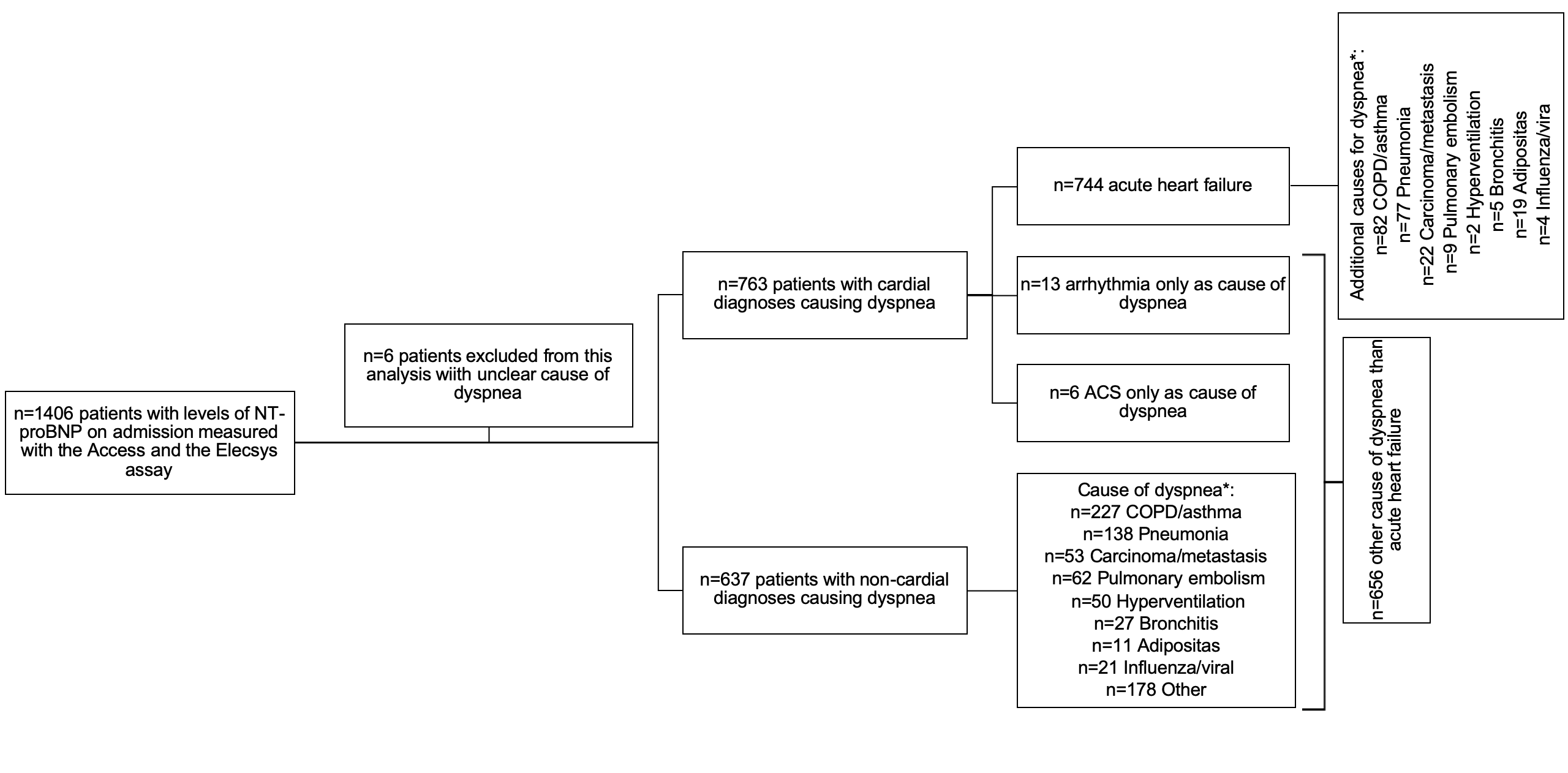


*Patients may have had several causes of dyspnea, as adjudicated diagnoses allowed for the presence of multiple contributing factors to dyspnea.

**
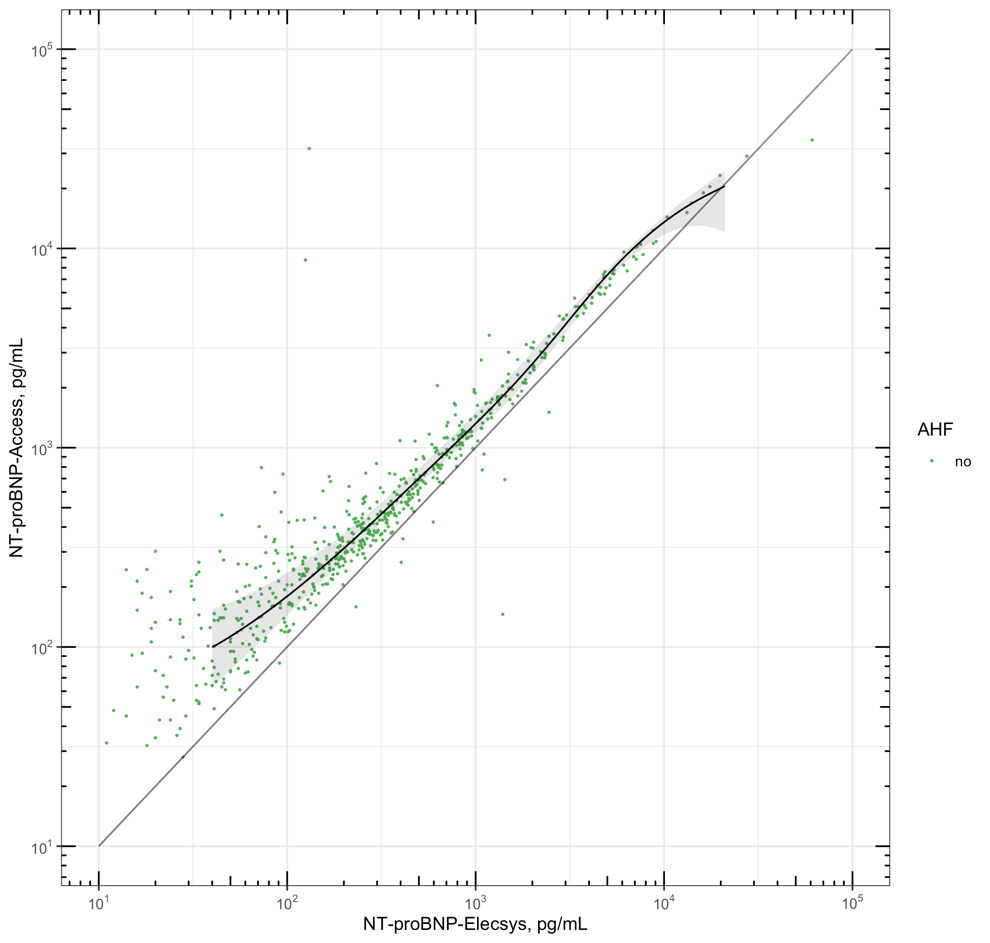

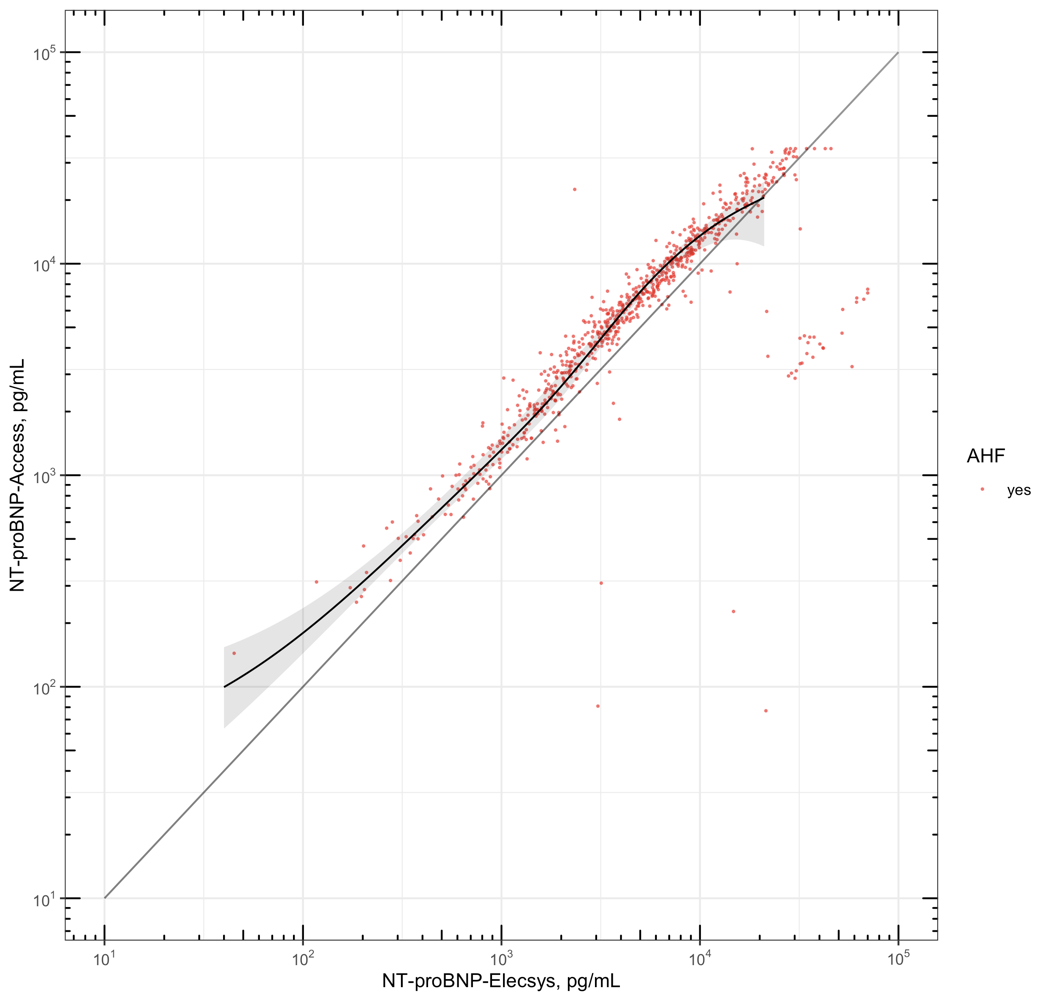
Supplemental Figure 2.** Direct comparison of both NT-proBNP assays in diagnostic subgroups (acute heart failure versus no acute heart failure).

**A** Quantile regression in patients without acute heart failure. **B** Quantile regression in patients with acute heart failure.

Quantile regression with restricted cubic splines using 5 knots. Red dots indicate patients with AHF, green dots patients without; grey diagonal represents x=y.

**C** Blant-Altman plot in patients without acute heart failure. **D** Blant-Altman plot in patients with acute heart failure.


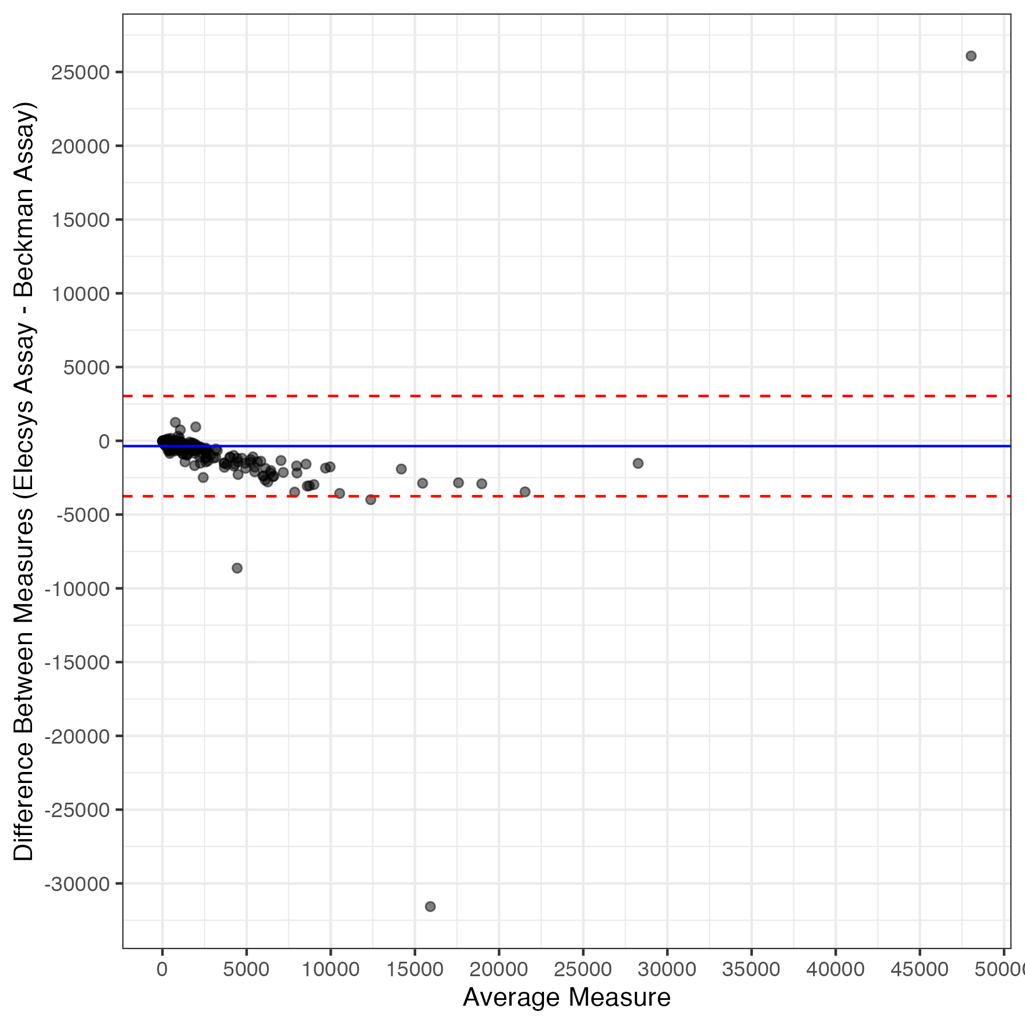

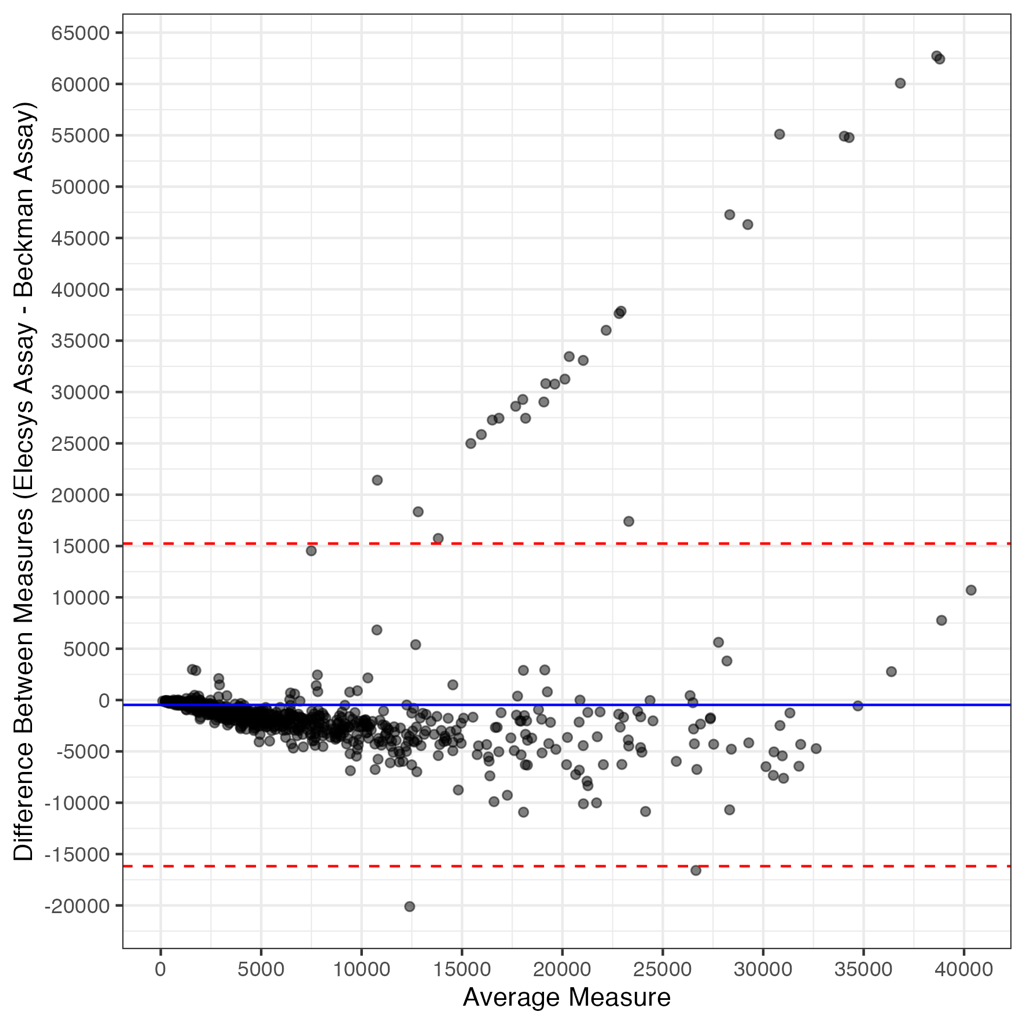


The X-axis represents the mean of NT-proBNP measurements (Access and Elecsys) for a pertaining patient. The Y-axis represents the difference between the NT-proBNP concentrations (Elecsys-Access). The blue line is mean of the difference between both assays. The red dashed lines are the upper and lower limits of agreement (mean of the difference +/- standard deviation of the difference*1.96). Patients are stratified according to final adjudicated diagnosis. AHF: acute heart failure. NT-proBNP: N-Terminal pro-B-type natriuretic peptide.

**Supplemental Figure 3.** Comparison of diagnostic accuracies between NT-proBNP-Access and NT-proBNP-Elecsys assay in predefined subgroups.


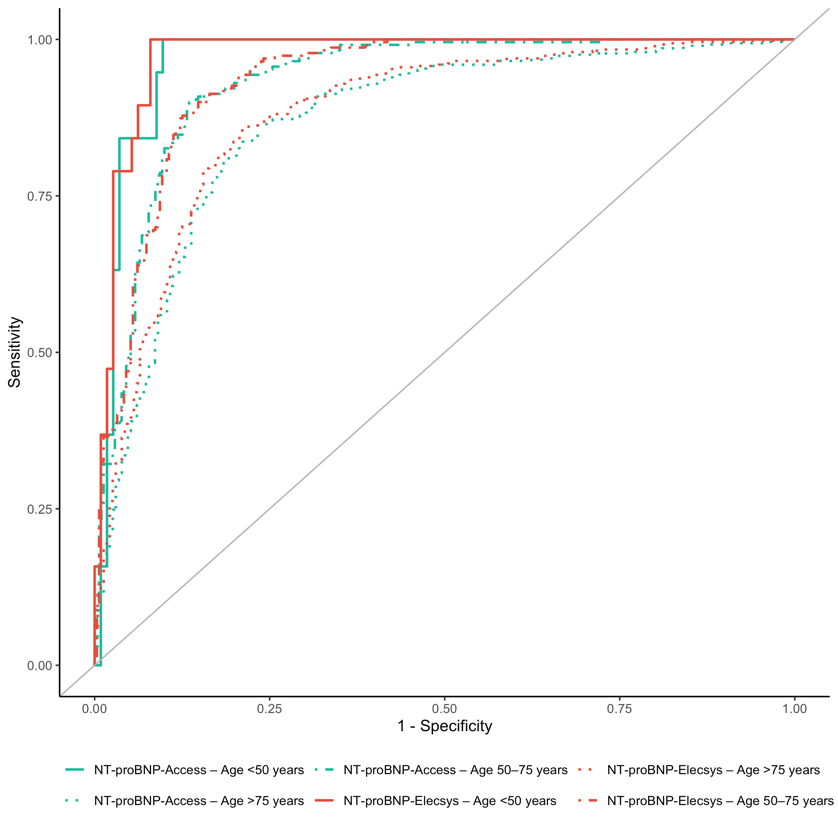

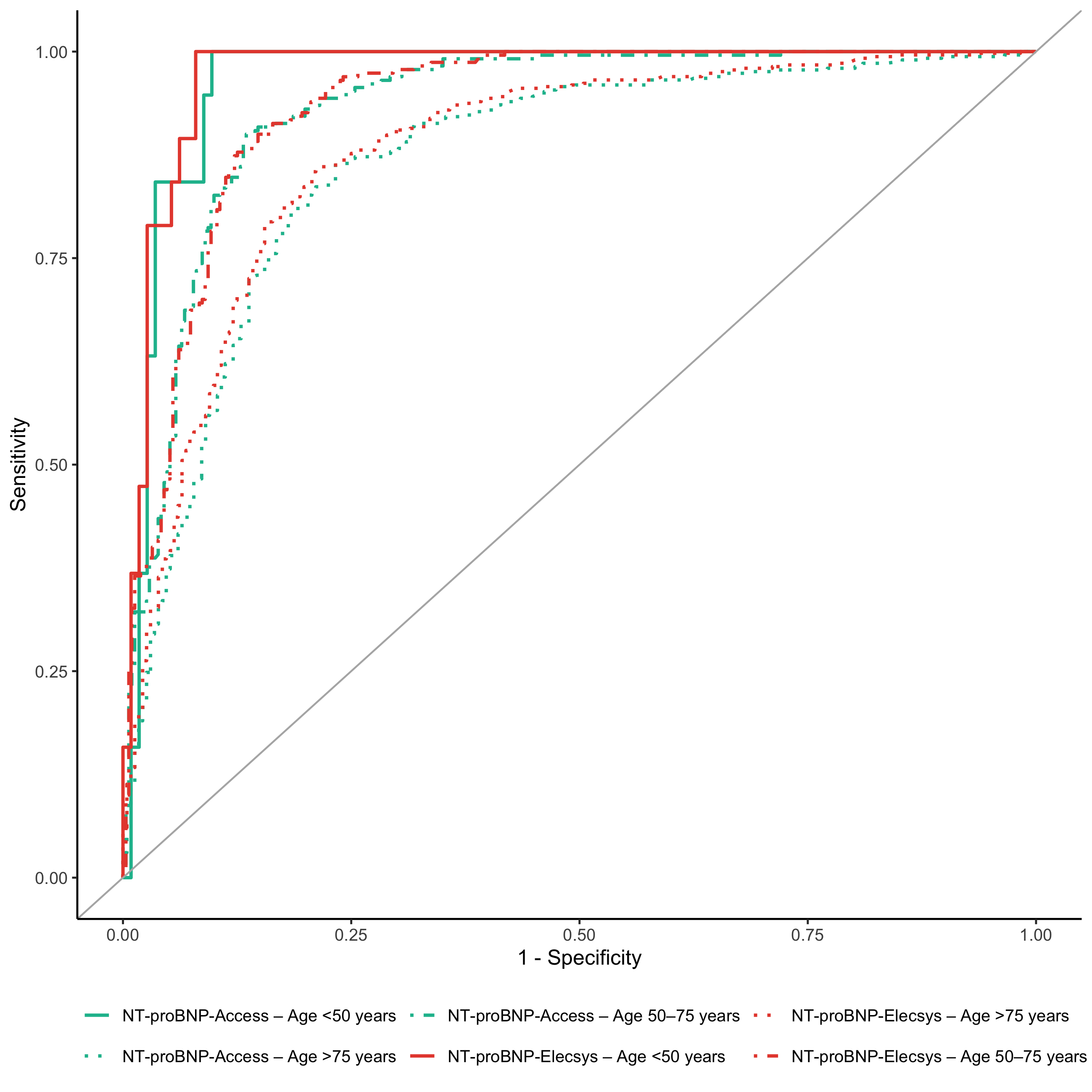

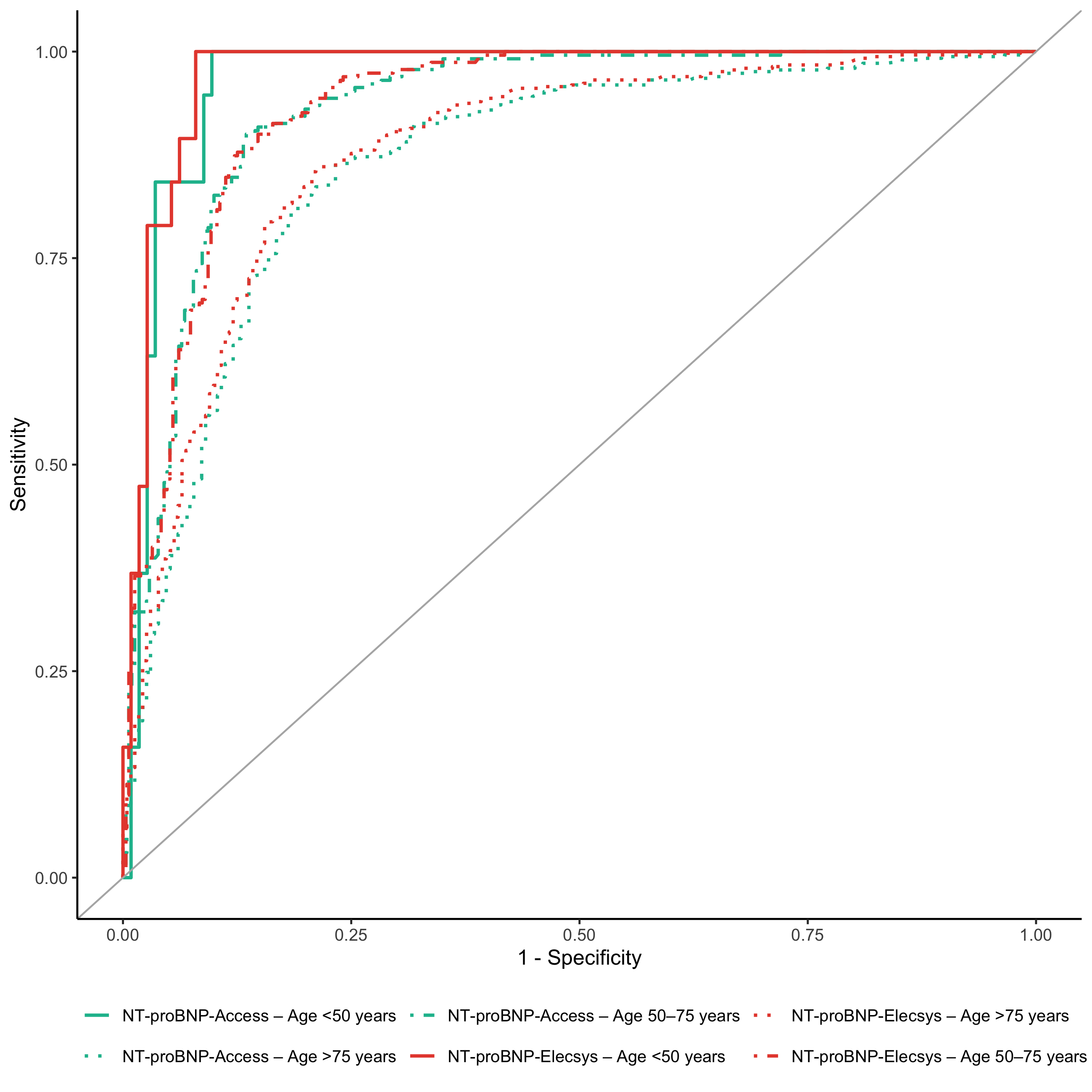

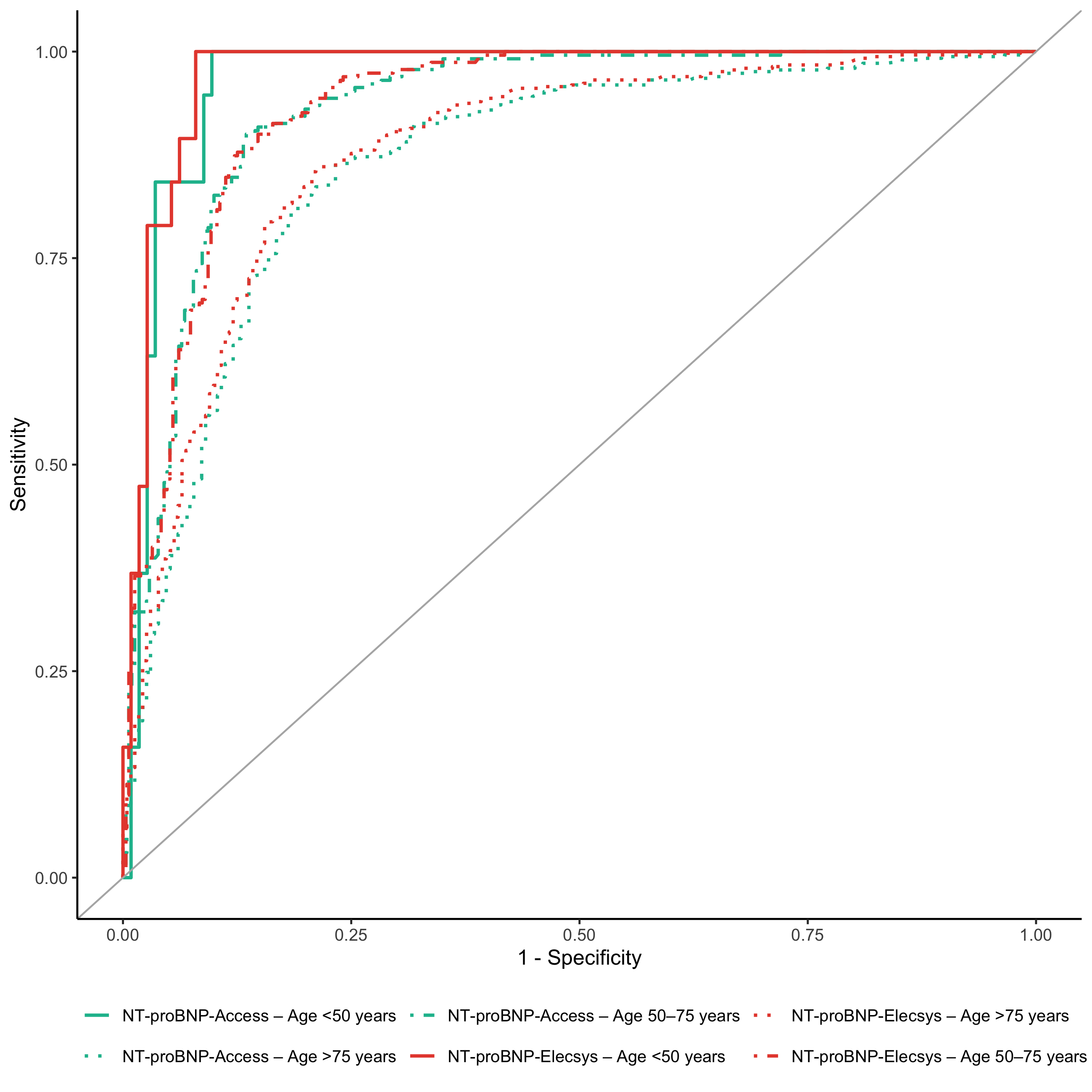


**
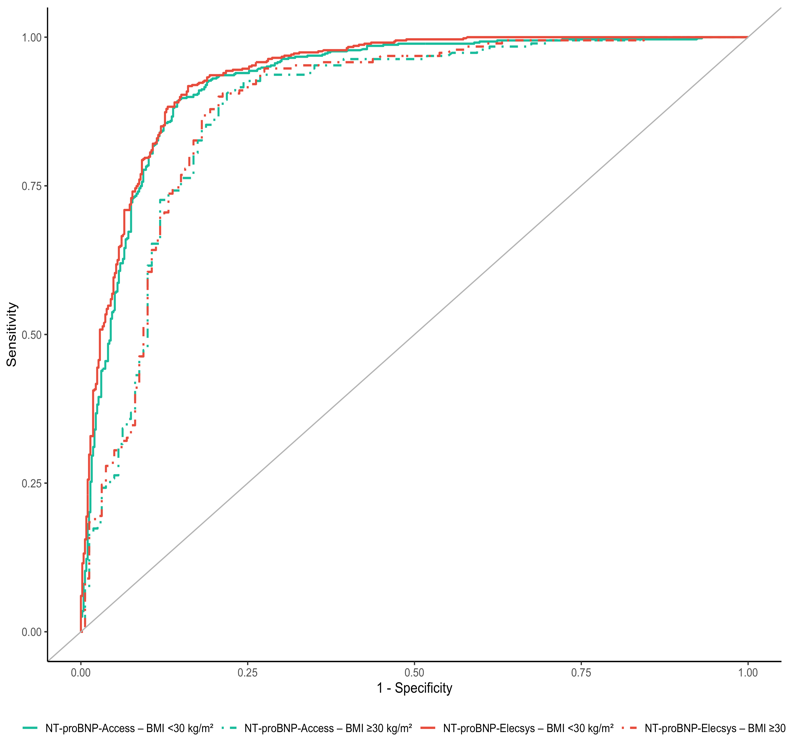

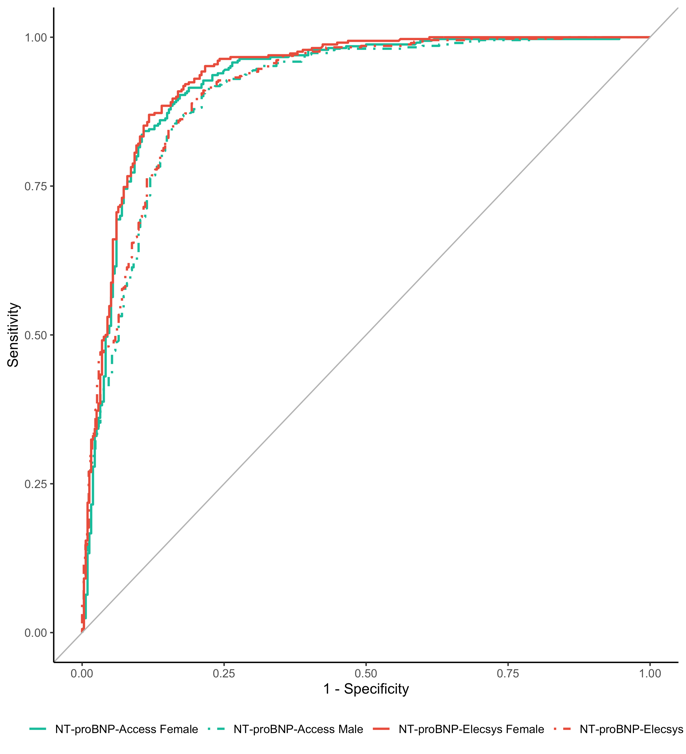
**

**
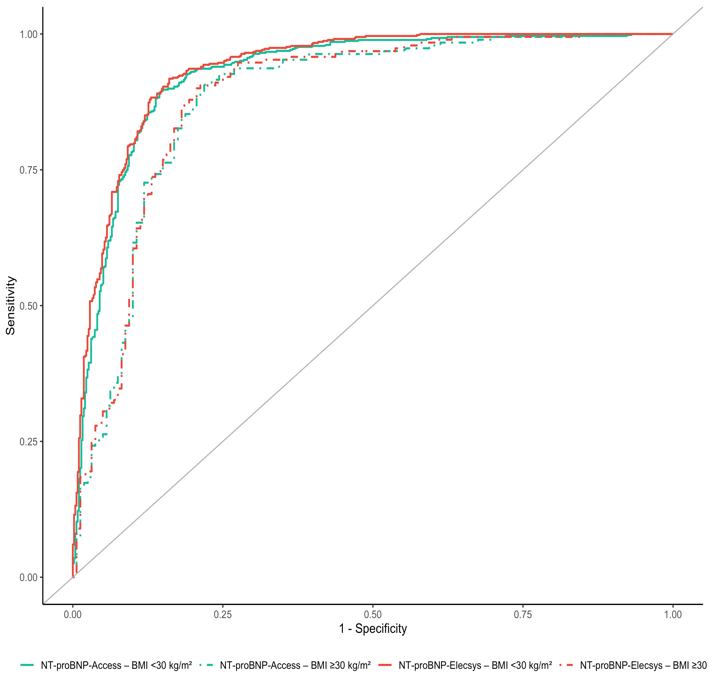
**


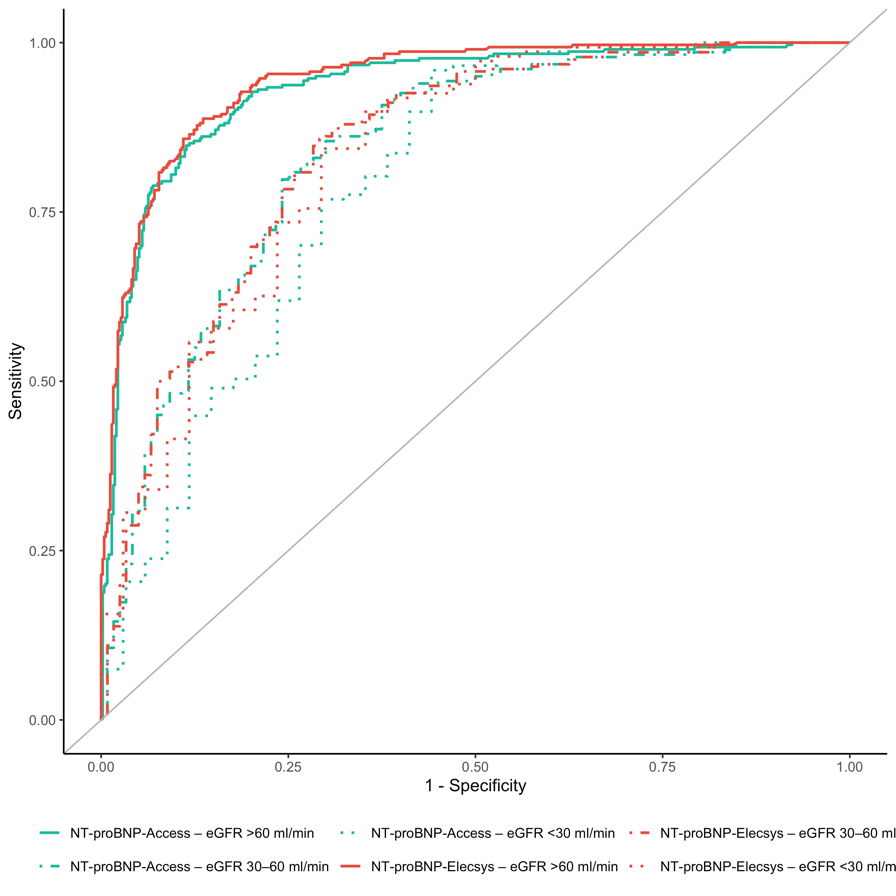

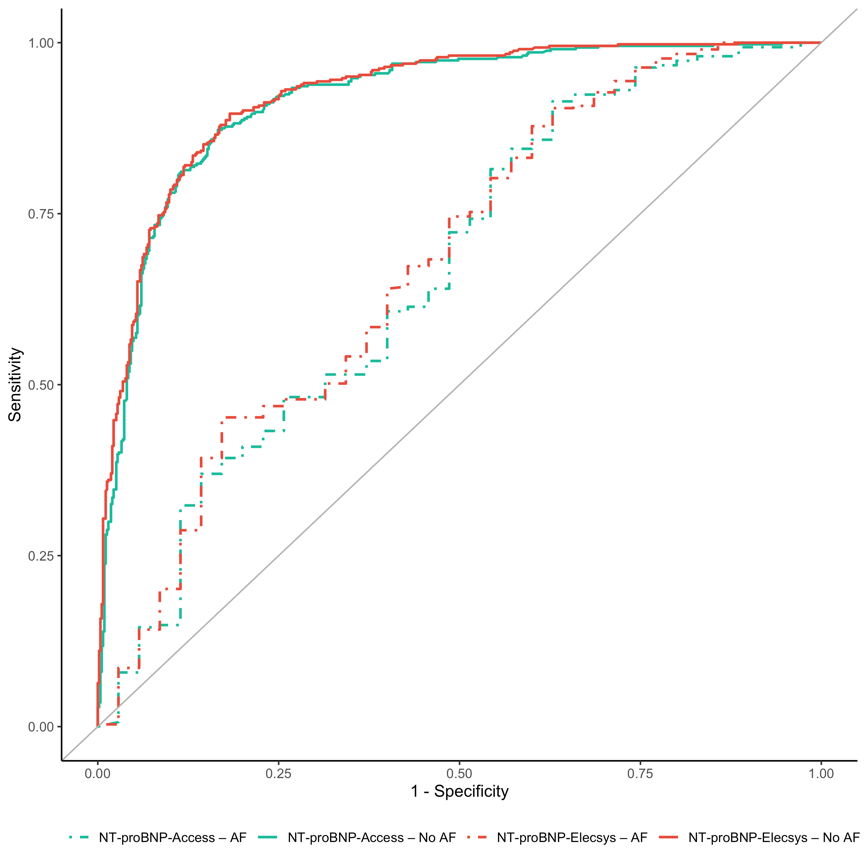
Clinical subgroups: sex, age (<50, 50–75, >75 years), body mass index (<30, ≥30 kg/m²), renal function (eGFR >60, 30–60, <30 ml/min/1.73 m²), and atrial fibrillation on admission.


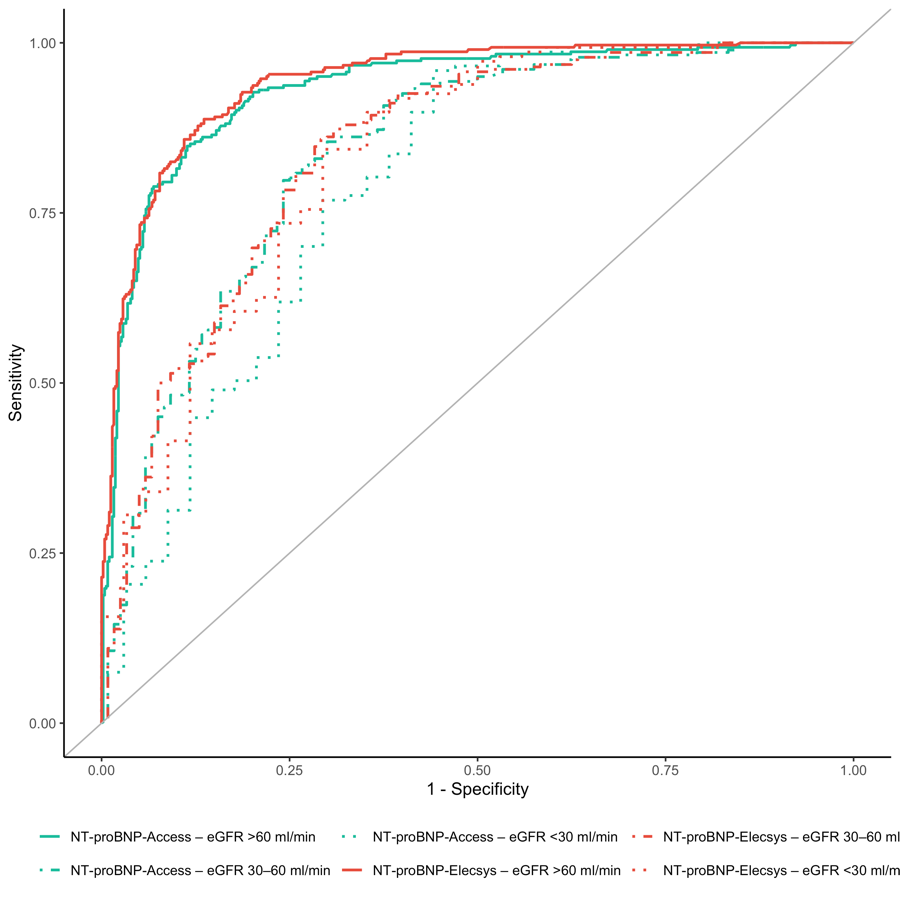

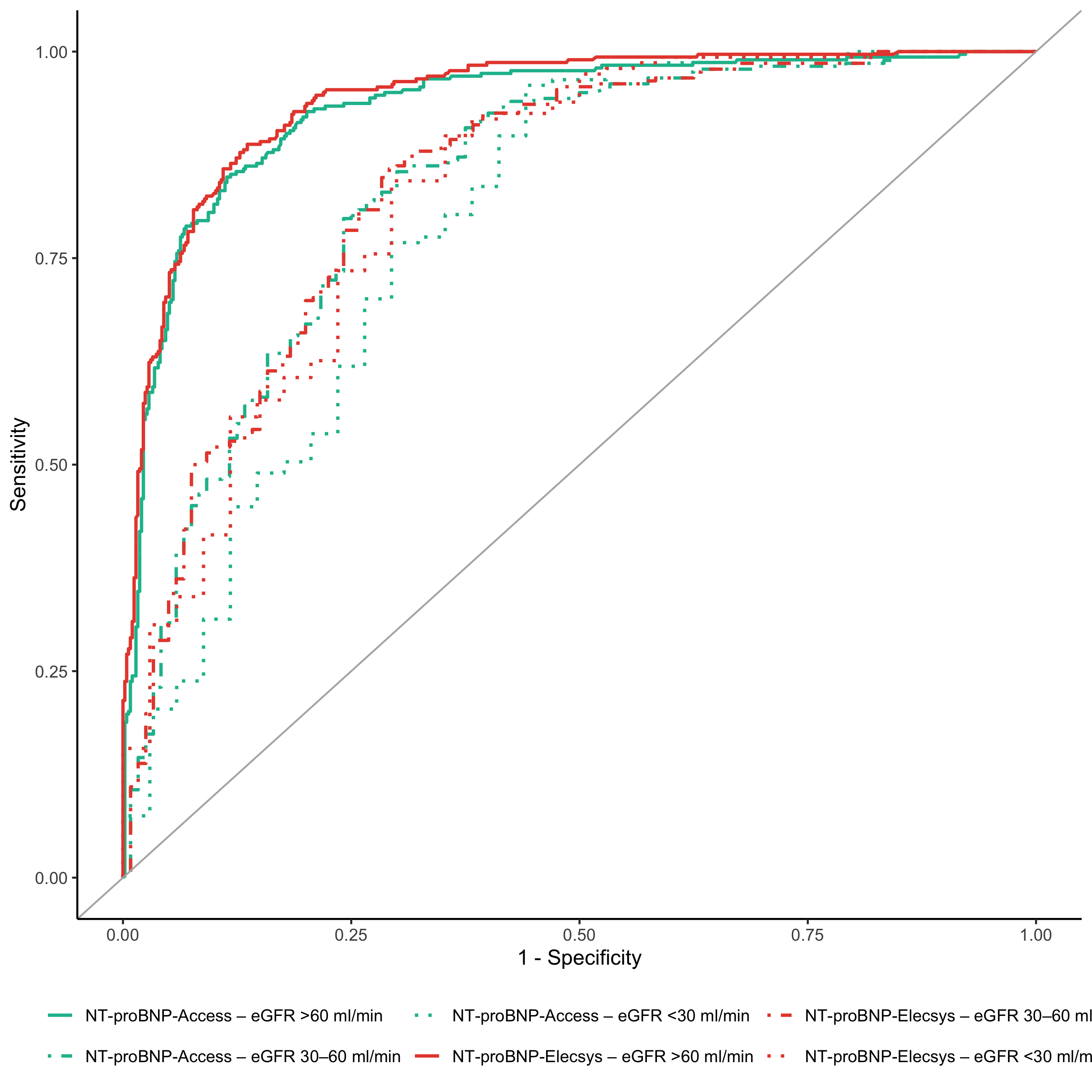

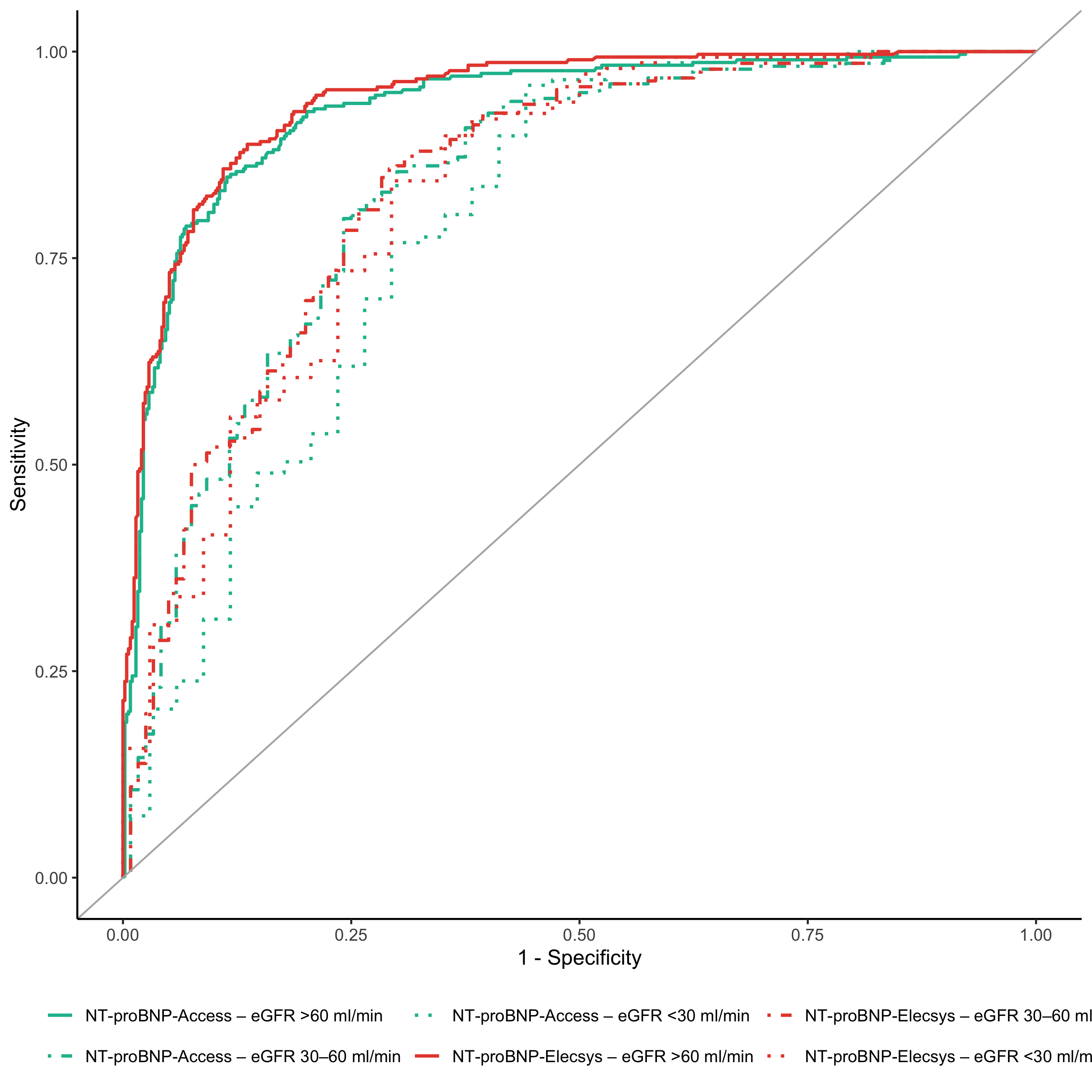

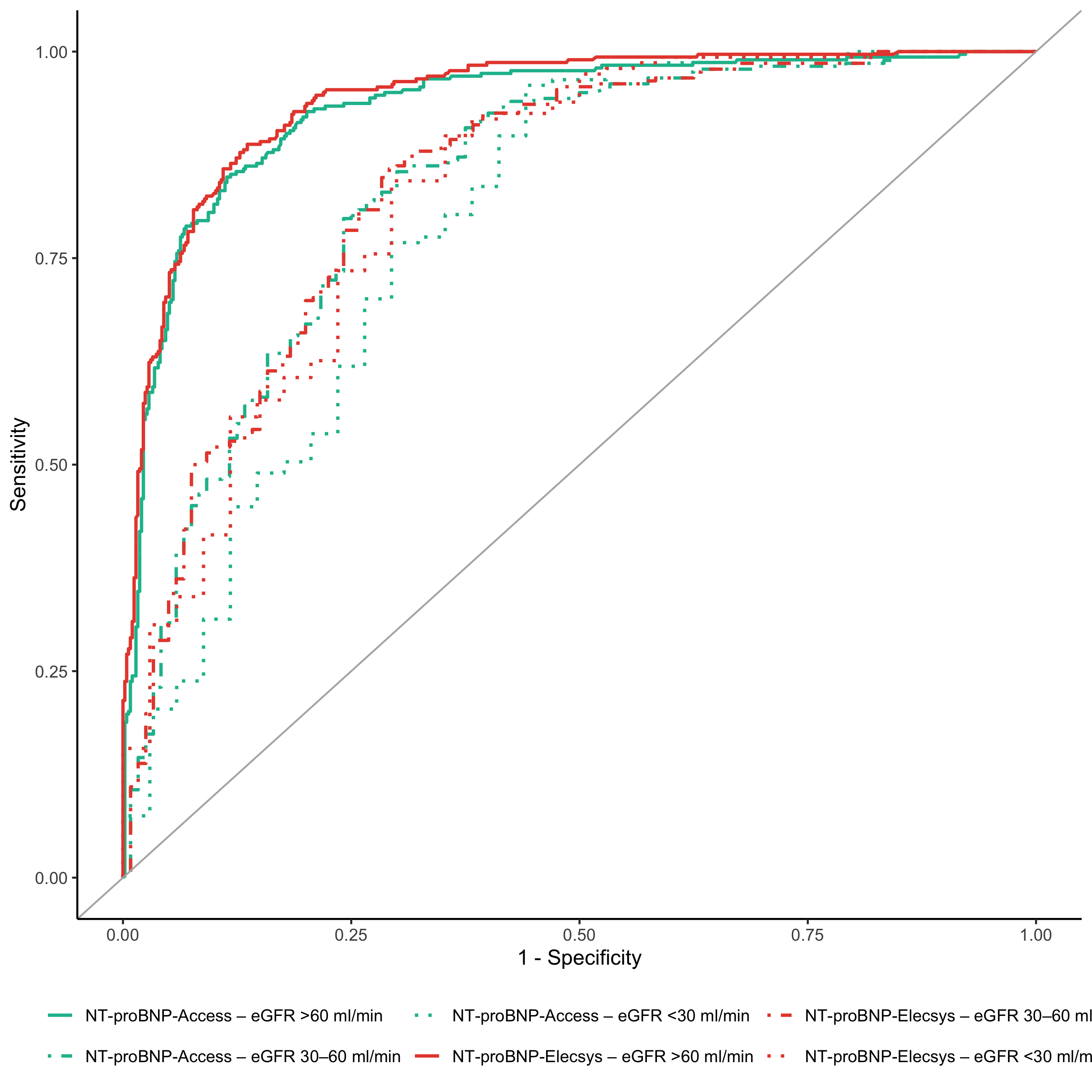


AF: atrial fibrillation. BMI: body mass index, eGFR: estimated glomerular filtration rate, NT-proBNP: N-terminal pro-B-type natriuretic peptide. eGFR was calculated using the Chronic Kidney Disease Epidemiology Collaboration formula.

**Supplemental Figure 4.** Comparison of diagnostic accuracy of NT-proBNP in diagnosing acute heart failure in obese patients.


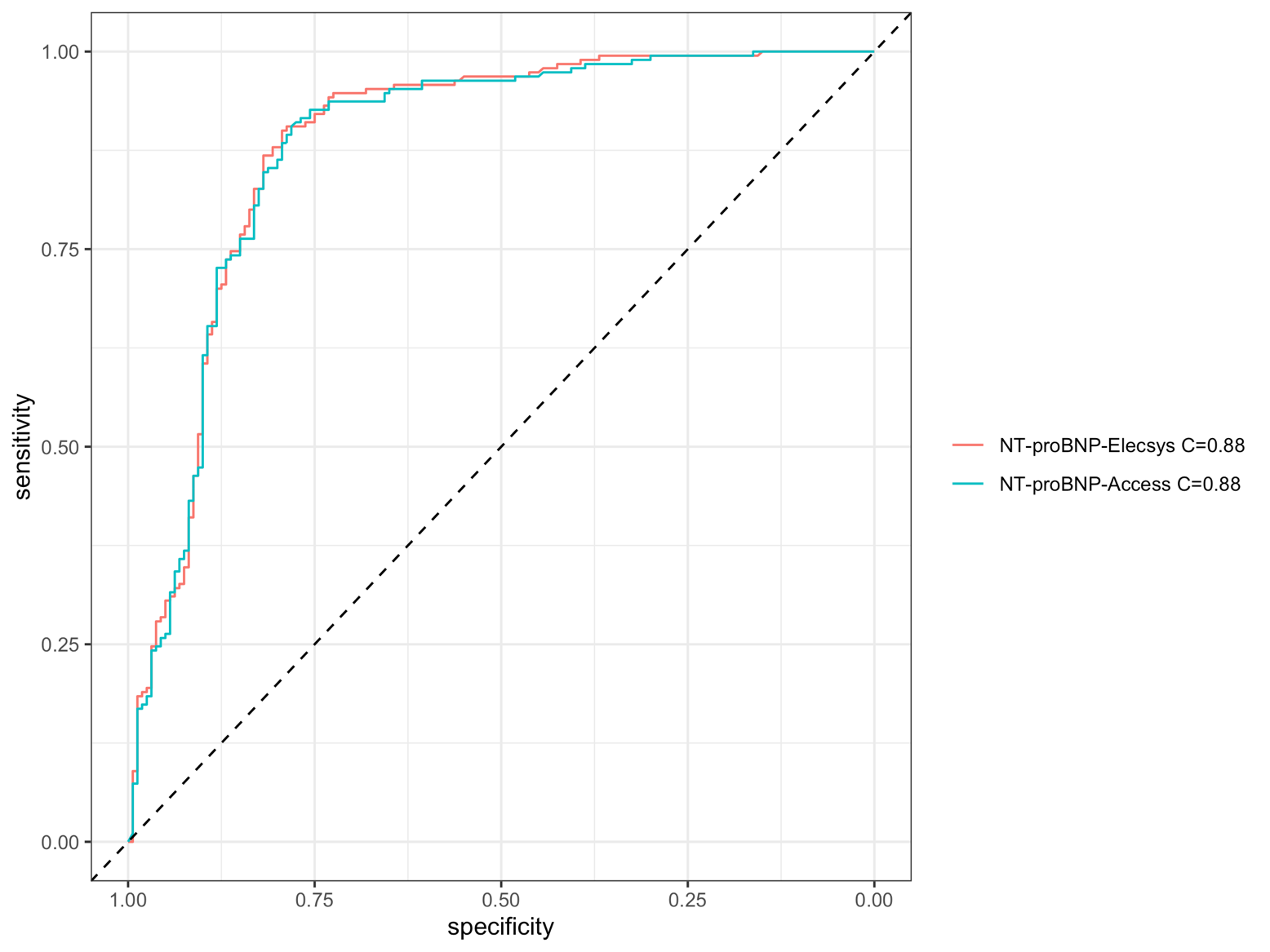


The Area under the receiver operating characteristics curve was 0.880 (CI 0.842 to 0.191) for the NT-proBNP Access and 0.884 (CI, 0.846 to 0.922) for the NT-proBNP Elecsys (DeLong test p-value = 0.163). AHF: acute heart failure. NT-proBNP: N-Terminal pro-B-type natriuretic peptide.

**Supplemental Figure 5.** Triage pathway to diagnose acute heart failure in obese patients.


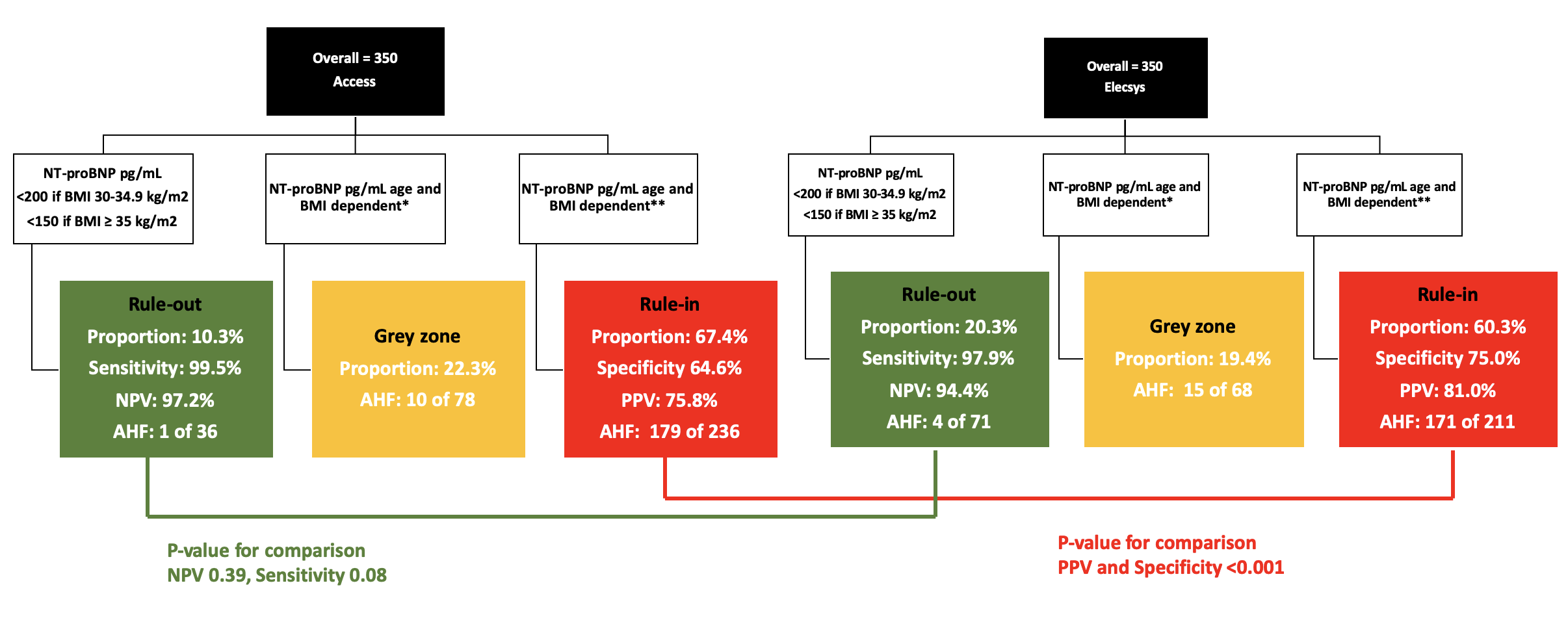


*If the BMI is 30–34.9 kg/m^2^, the NT‐proBNP rule‐in cut‐off is 300 ng/L for patients <50 years old, 600 ng/L for patients 50–75 years old, and 1200 ng/L for patients >75 years old. If the BMI is ≥35 kg/m^2^, the NT‐proBNP rule‐in cut‐off is 225 ng/L for patients <50 years old, 450 ng/L for patients 50–75 years old, and 900 ng/L for patients >75 years old. AHF: acute heart failure. NPV: negative predictive value. NT-proBNP: N-Terminal pro-B-type natriuretic peptide. PPV: positive predictive value


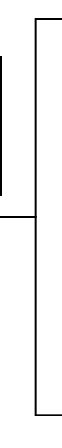
 **Supplemental Figure 6.** Diagnostic concordance between NT-proBNP-Access and NT-proBNP-Elecsys in obese patients.

NT-proBNP-Access


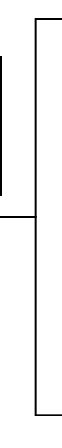

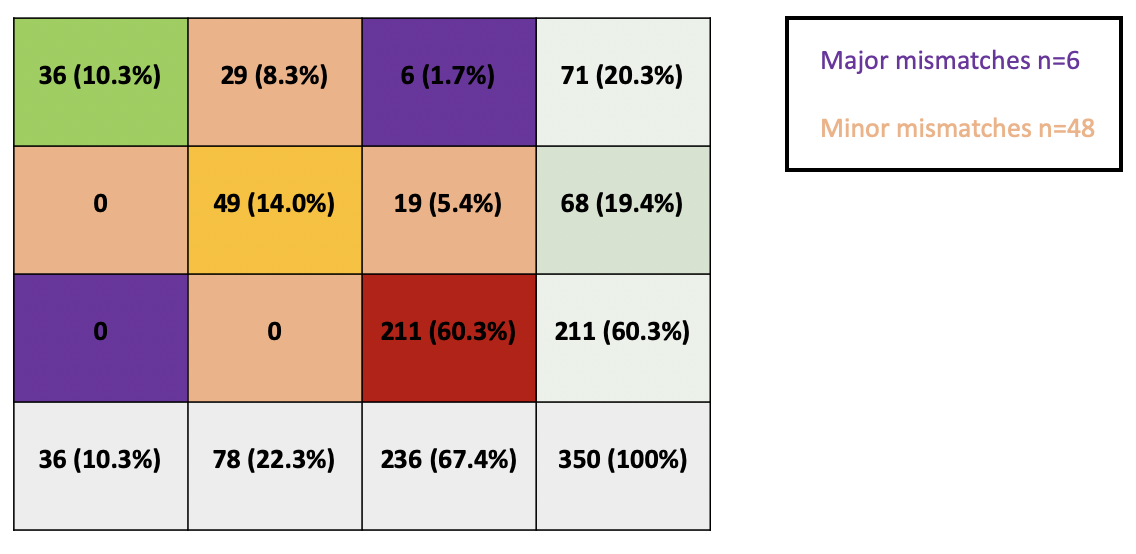


rule-in observe rule-out

NT-proBNP-Elecsys

rule-out observe rule-in

A disagreement was considered major (lilac) in those cases where triage decision for one assay was rule-out (red) while for the other one it was rule-in (green). Minor disagreements (nude) were following: rule-out vs grey zone (yellow) or grey zone vs rule-in. These values represent differences in triage categorization (rule-in, rule-out, or grey zone) between assays and do not correspond to false or true positives. Data is represented as number (percent)


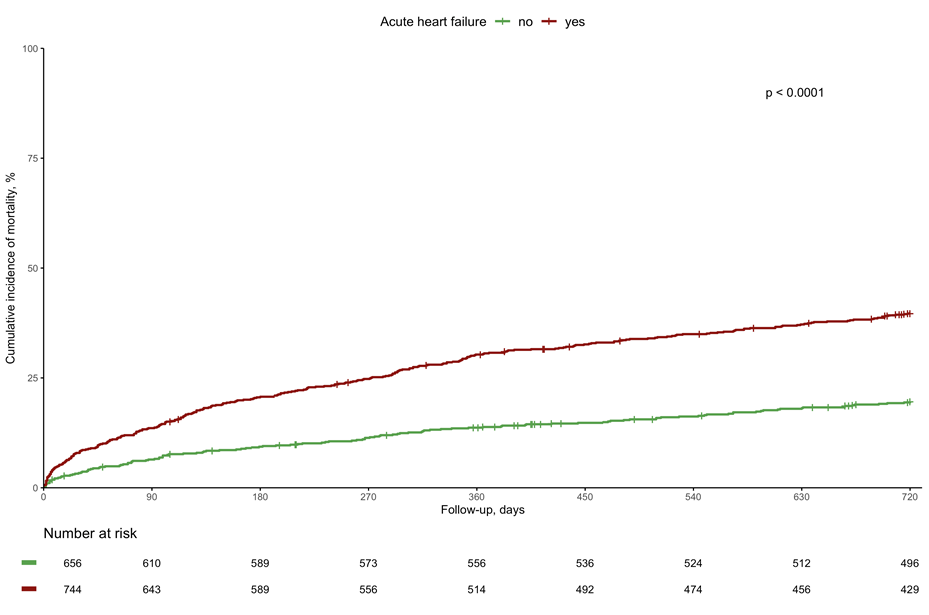
**Supplemental Figure 7.** All-cause mortality within 2 years of follow up in patients with acute heart failure and in patients with other causes of acute dyspnea.

Kaplan-Meier survival curve displaying the cumulative incidence of all-cause mortality over a follow-up period of 720 days, comparing two groups of patients based on the presence or absence of acute heart failure (AHF) as adjudicated diagnosis for dyspnea. **Red line**: Patients with AHF. **Green line**: Patients without AHF.

AHF: acute heart failure. p-value: log rank test.

**Supplemental Figure 8.** Comparison of prognostic accuracies of NT-proBNP-Elecsys and NT-proBNP-Access **A** within 720 days of follow-up for all-cause mortality, for **B** all-cause mortality at 360 days and for **C** all-cause mortality at 720 days.


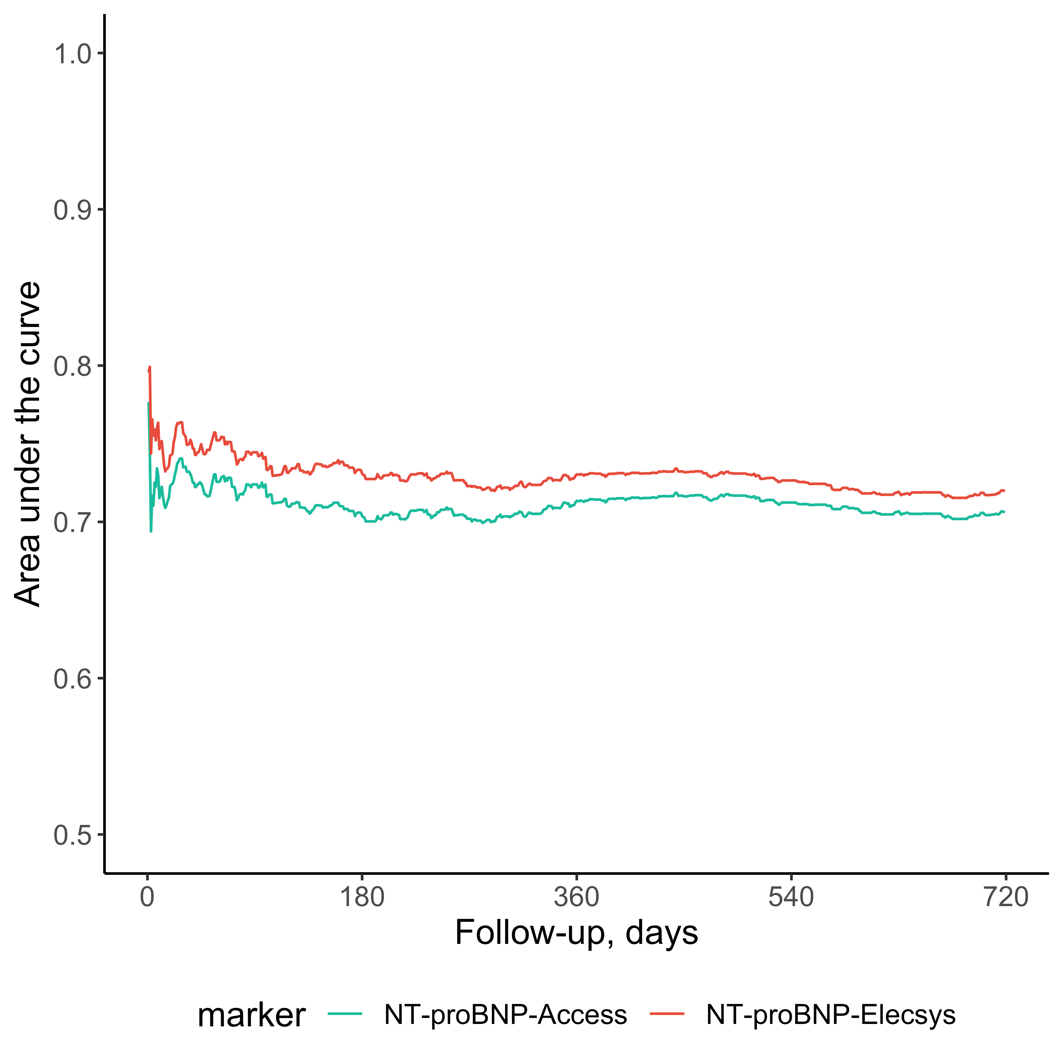


**A**

**B**


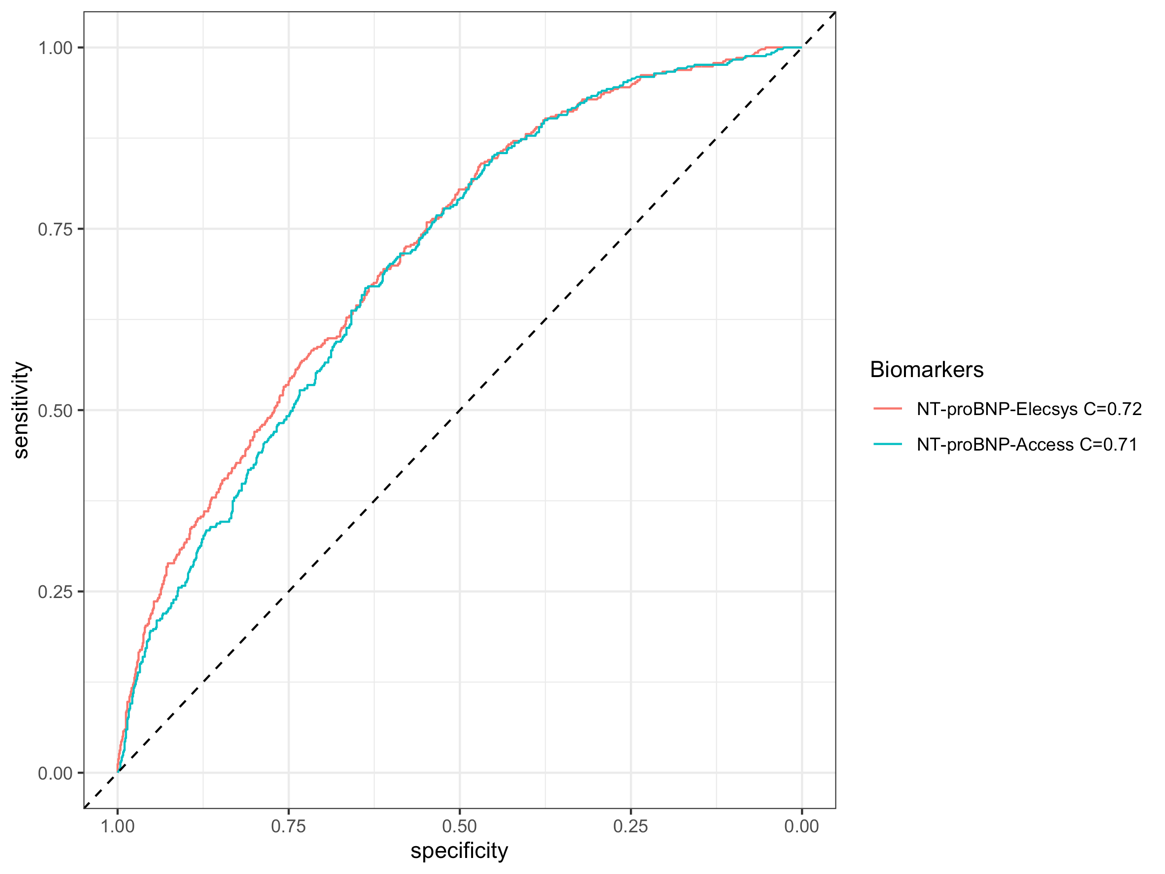
**
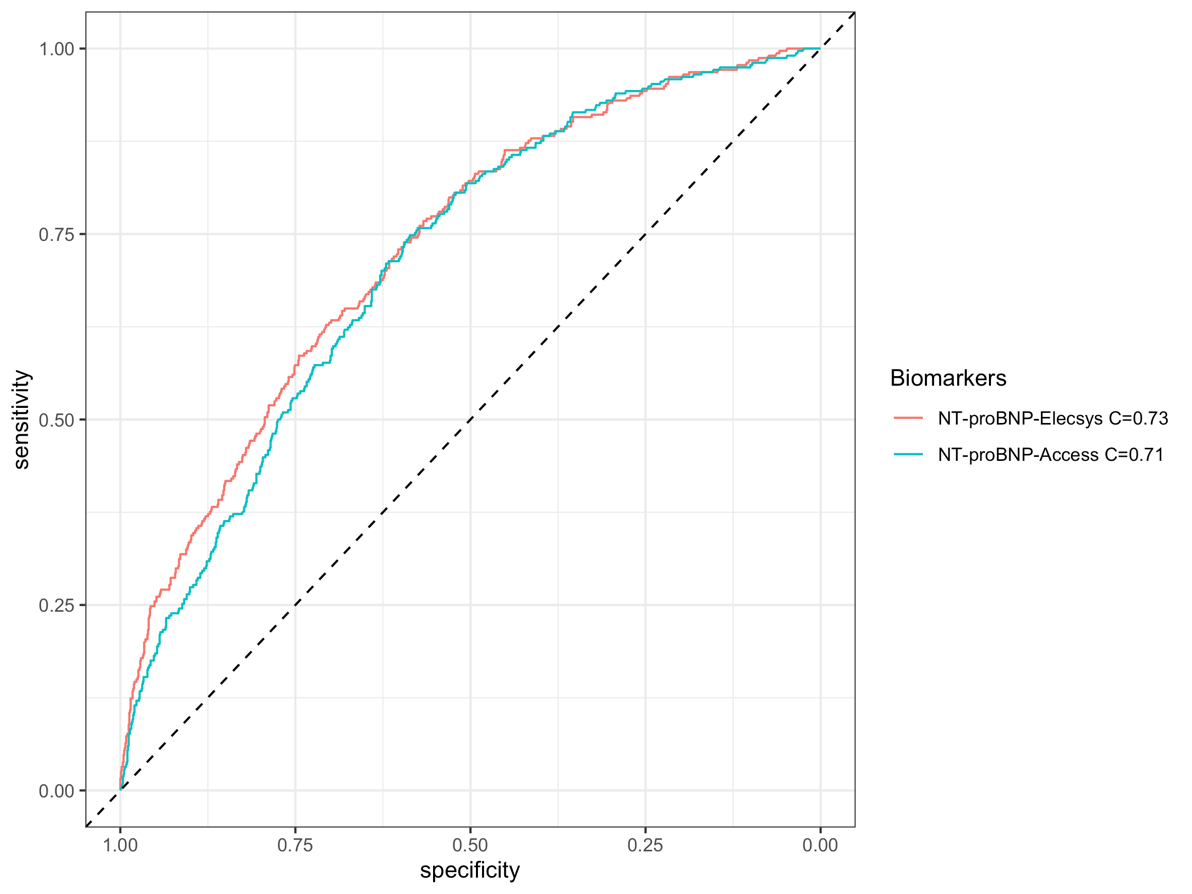
**

**C**
